# DiCleavePlus: A Transformer‐Based Model to Detect Human Dicer Cleavage Sites Within Cleavage Patterns

**DOI:** 10.1111/gtc.70074

**Published:** 2025-12-14

**Authors:** Lixuan Mu, Tatsuya Akutsu

**Affiliations:** ^1^ Bioinformatics Center, Institute for Chemical Research, Kyoto University Kyoto Japan

**Keywords:** attention‐based neural network, deep learning, dicer cleavage site prediction, miRNA

## Abstract

MicroRNAs (miRNAs) play a crucial role in posttranscriptional gene regulation. The biogenesis of mature miRNAs requires precise cleavage of precursor miRNAs (pre‐miRNAs) by Dicer. Several computational approaches have been developed to predict human Dicer cleavage sites; however, important limitations persist. Cleavage Pattern‐based models, which rely on short pre‐miRNA subsequences, can only identify positive patterns in which the cleavage site is centrally located. Conversely, models that do not rely on Cleavage Patterns generally exhibit suboptimal performance. These limitations highlight the need for a more accurate predictor that fully exploits sequence and structural information from pre‐miRNAs. In this study, we propose DiCleavePlus, a Cleavage Pattern‐based framework for predicting Dicer cleavage sites on pre‐miRNAs. DiCleavePlus takes an extended Cleavage Pattern together with the full‐length pre‐miRNA sequence from which it is derived as input. A Transformer‐based encoder is employed to extract features from both the pattern and the pre‐miRNA. Benchmarking experiments demonstrate that DiCleavePlus achieves accurate and robust performance in predicting human Dicer cleavage sites.

## Graphical abstract

DiCleavePlus integrates pre‐miRNA sequence and secondary structure information through Transformer‐based feature extraction to predict Dicer cleavage site positions with high accuracy. The model converts Dicer cleavage prediction into a multi‐class task, enabling precise and robust identification of cleavage intervals across human pre‐miRNAs.

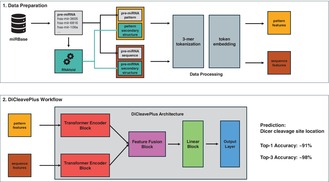

## 1 Introduction

MicroRNAs (miRNAs) are a class of single‐stranded, noncoding RNAs that play critical roles in posttranscriptional gene regulation through RNA silencing (Lee et al. 1993; Wightman et al. 1993; Hammond et al. 2000; Yoshida et al. 2021). Dysfunction of miRNAs has been implicated in various human diseases, including cancers (Calin et al. 2002; Takamizawa et al. 2004; Lu et al. 2005; Yu et al. 2007; Ali Syeda et al. 2020), cardiovascular diseases (Zhao et al. 2005; Care et al. 2007; Thum et al. 2008; Small and Olson 2011; Kalayinia et al. 2021), autoimmune diseases (Taganov et al. 2006; Tang et al. 2009; Pauley et al. 2009; Yan et al. 2019), and infectious diseases (Huang et al. 2007; Skalsky and Cullen 2010; Kimura et al. 2023). In the canonical miRNA biogenesis pathway, primary miRNAs (pri‐miRNAs) are transcribed in the nucleus (O'Brien et al. 2018). Drosha, an RNase III enzyme, cleaves the flanking regions of pri‐miRNAs to generate precursor miRNAs (pre‐miRNAs) (Yoshida et al. 2021; Nguyen et al. 2015). Pre‐miRNAs are exported to the cytoplasm and further cleaved by Dicer, another RNase III enzyme, to produce mature miRNAs (O'Brien et al. 2018).

Dicer is a key RNase III enzyme essential for the proper maturation of miRNAs (Kim et al. 2009; Ha and Kim 2014; Le et al. 2024). It contains multiple functional domains (Lau et al. 2012). Among these the RNase IIIa and RNase IIIb domains are pivotal for cleaving precursor RNA molecules (Nguyen et al. 2022; Le et al. 2024), while the PAZ domain participates in determining cleavage positions (Lau et al. 2012; Nguyen et al. 2022). Since miRNAs regulate gene expression through base pairing with target messenger RNAs (mRNAs), precise Dicer cleavage is of importance; for example, inaccurate cleavage can generate miRNAs with altered seed regions (Lewis et al. 2003), thereby impacting the proper function of miRNAs. Experimental identification of Dicer cleavage sites in relation to pre‐miRNA sequence and secondary structure relies on techniques such as Northern blotting, primer extension, isotopic or chemical labeling, in vitro RNA synthesis, RNA immunoprecipitation, and high‐throughput RNA sequencing (Hutvagner et al. 2001; Zhang et al. 2004; Vermeulen et al. 2005; Starega‐Roslan et al. 2015; Luo et al. 2021; Nguyen et al. 2022). These methods, however, are labor‐intensive, time‐consuming, and costly. Therefore, an accurate computational predictive model of Dicer cleavage sites can greatly facilitate experimental design and biological discovery.

**FIGURE 1:**
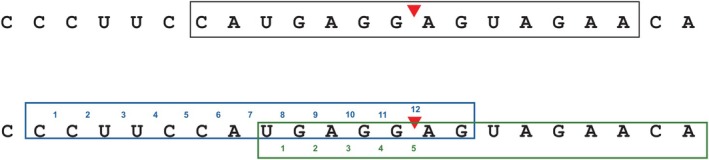
Concept of Cleavage Pattern. Conventional Cleavage Patterns (upper, black frame) restrict the cleavage site (red triangle) to the central interval. In DiCleavePlus, Cleavage Patterns are generated using a sliding window approach along the pre‐miRNA sequence, allowing the cleavage sites to appear at any interval within the pattern (lower, blue and green frames). For example, the blue pattern is labeled as class 12, while the green pattern is labeled as Class 5.

Several machine learning models have been developed for predicting human Dicer cleavage sites. PHDcleav (Ahmed et al. 2013) and LBSizeCleav (Bao et al. 2016) employed support vector machines. Later, Liu et al. (2021) proposed a gradient boosting‐based model, ReCGBM, which improved prediction precision. Given the prevalence of deep learning, Mu et al. (2024) proposed a deep learning model named DiCleave and achieved performance comparable to ReCGBM. These methods introduced the concept of Cleavage Pattern, which is typically a 14‐nucleotide‐long subsequence extracted from a pre‐miRNA sequence (Figure 1). Cleavage Pattern‐based models are trained to predict whether a given pattern contains a Dicer cleavage site. In contrast, DeepMirCut (Bell and Hendrix 2022), an RNN‐based model that does not rely on Cleavage Patterns, directly predicts both Drosha and Dicer cleavage sites from full‐length pre‐miRNA sequences. However, its prediction accuracy remains suboptimal.

Although Cleavage Pattern‐based models demonstrate superior performance, they have a critical limitation: they are restricted to detecting Dicer cleavage sites only when they occur at the central position of the patterns (Figure 1, upper, black frame). For instance, the human pre‐miRNA has‐let‐7a‐1, which consists of 80 nucleotides (nt), can generate 67 Cleavage Patterns when the pattern length is set to 14 nt (Figure S1). Among these, 26 patterns actually contain a Dicer cleavage site, yet existing models can identify only 2 patterns where the cleavage site lies at the center (Figure S1, red frames). Consequently, these models oversimplify the prediction as a binary classification task (i.e., whether a pattern contains a cleavage site), without fully exploring the positional diversity of cleavage sites. Moreover, despite reporting binary accuracies around 0.90, these models still face a non‐negligible risk of misidentifying true cleavage sites. Therefore, there remains considerable scope for improving Dicer cleavage site prediction.

Transformer (Vaswani et al. 2017) applies the multi‐head attention mechanism to alleviate the gradient vanishing problem associated with long input sequences. With its success in natural language processing, Transformer has become a popular architecture in bioinformatics (Zhang et al. 2023). Recent works by Yao et al. (2024), Li et al. (2024), and Zhang et al. (2024) employed the encoder part of the Transformer as feature extractors to exploit hidden features from input data, taking advantage of the strong capacity to capture internal relationships.

In this study, we propose DiCleavePlus, a Transformer‐based model for human Dicer cleavage site prediction. Compared with previous works, DiCleavePlus extends Cleavage Pattern by allowing cleavage sites to appear at any position within the pattern, which maximizes the informational content extracted from pre‐miRNA sequences. DiCleavePlus incorporates both the extended Cleavage Pattern and the corresponding full‐length pre‐miRNA as joint inputs and employs Transformer encoders to extract features from both sequence and secondary structure representations. Benchmarking experiments demonstrate that DiCleavePlus achieves high accuracy and robustness in cleavage site prediction. By allowing flexible cleavage site positioning and integrating multi‐level RNA information, DiCleavePlus maximizes the utility of pre‐miRNA features. These improvements highlight its potential as a practical tool for guiding experimental design, narrowing the search space of candidate sites, and ultimately facilitating downstream studies of miRNA biogenesis and function.

## 2 Results

### 2.1 Overview of the Framework

#### 2.1.1 Extended Cleavage Pattern

A conventional Cleavage Pattern is defined as a short subsequence of a pre‐miRNA sequence (Ahmed et al. 2013; Bao et al. 2016; Liu et al. 2021; Mu et al. 2024). Patterns in which the cleavage site is located at the central position are considered positive, whereas those that do not contain cleavage site are labeled negative. Typically, a Cleavage Pattern is 14 nucleotides in length, resulting in 13 intervals between adjacent nucleotides (Figure 1). Previous models based on conventional Cleavage Patterns can detect only one type of positive pattern where the cleavage site is positioned at the central interval (Figure 1, upper, black frame).

To overcome this limitation, we expanded the definition of positive Cleavage Patterns by allowing the cleavage sites to appear at any interval within the pattern (Figure 1, lower, blue and green frames). In this study, each interval index is regarded as a unique class. Specifically, Class 0 represents negative patterns (i.e., no cleavage site), while Classes 1–*k* correspond to cleavage sites located at interval positions within the pattern.

With this extension, the Dicer cleavage site prediction task is converted into a multi‐class classification problem, where the model predicts the index of the interval containing the cleavage site or assigns Class 0 if no cleavage site is present. To investigate the impact of pattern size on model performance, Cleavage Patterns of 10, 12, 14, 16, and 18 nt were generated and used in subsequent experiments (see Section 4).

#### 2.1.2 Model Architecture

**FIGURE 2:**
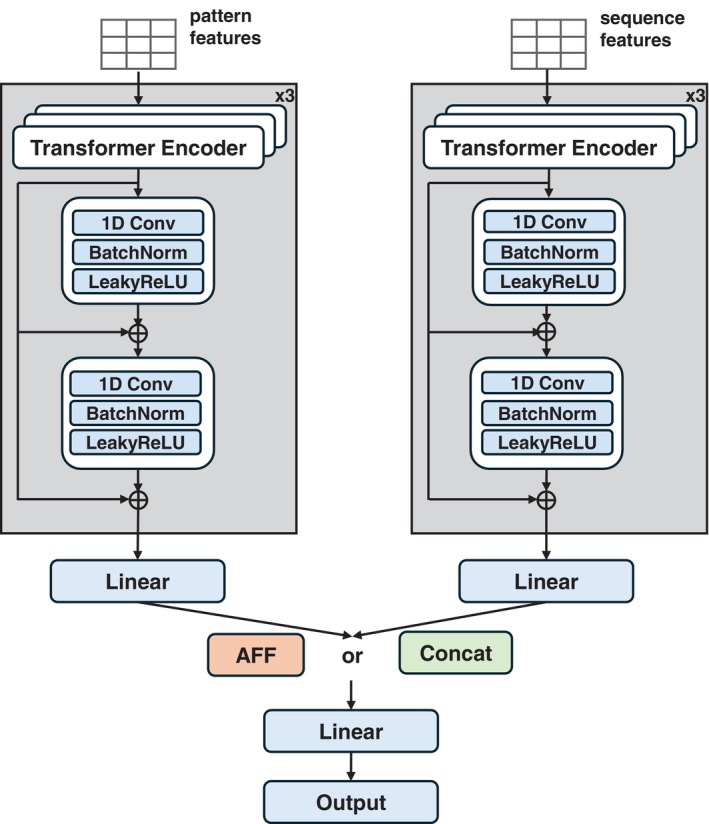
Overview of the DiCleavePlus architecture. The gray modules represent the Transformer‐based Encoder Units, which are used to extract features from Cleavage Patterns and full‐length pre‐miRNA sequences. Two feature integration strategies were implemented. Models employing the attentional feature fusion (AFF) block to integrate processed pattern and sequence features are referred to as DCP‐AFF, whereas models that merge these features using simple concatenation are referred to as DCP‐Concat.

The overall architecture of DiCleavePlus is illustrated in Figure 2. DiCleavePlus utilizes Transformer‐based Encoder Units to process Cleavage Pattern features and full‐length pre‐miRNA sequence features. Each Encoder Unit begins with three standard Transformer encoder layers, each consisting of a multi‐head self‐attention layer followed by a feed‐forward layer (Vaswani et al. 2017). Following the Transformer layers, two convolutional blocks are applied to further refine the representations. Each block is composed of a one‐dimensional convolutional layer followed by a leaky ReLU activation layer (Maas et al. 2013). Residual connections (He et al. 2016) are added between the outputs of the Transformer layer and each convolutional block to facilitate gradient flow and preserve feature information. A final linear layer is applied to project the processed features into a fixed‐dimensional representation.

To integrate the processed pattern features and pre‐miRNA sequence features, we designed two fusion strategies. In the first strategy, we implemented the attentional feature fusion (AFF) module from Dai et al. (2021) to integrate these features. Details of the AFF module are provided in Section 4. Models that used this strategy are hereafter denoted as DCP‐AFF. In the alternative strategy, we employed a concatenation operation to merge pattern features and pre‐miRNA features. Models adopting this strategy are referred to as DCP‐Concat.

After feature fusion, the combined representation is passed through a fully connected block and then fed into the output layer. A softmax function is applied to predict the index of the interval where the Dicer cleavage site is located in the Cleavage Pattern (or Class 0 for negative patterns).

### 2.2 Performance of Cleavage Site Position Prediction

Two types of datasets were constructed from the miRBase database (Release 22.1; Griffiths‐Jones et al. 2007). Dataset‐1 reflects a realistic data distribution, where negative patterns constitute the majority class. Dataset‐2, in contrast, is class‐balanced, with an equal number of samples for each class. Details of data preparation and dataset construction are provided in Section 4.

We evaluated the performance of DiCleavePlus using fivefold cross‐validation on both datasets and across different pattern sizes. As shown in Table 1A, the performance of DCP‐AFF and DCP‐Concat improved consistently as the pattern size increased. When using the empirical 14‐nt pattern size on Dataset‐1, both models exhibited similar performance. Although the top‐1 accuracy was approximately 0.86, the top‐3 accuracy was remarkably high (DCP‐AFF: 0.979 and DCP‐Concat: 0.980), suggesting high practical reliability. Both models also achieved high perfect match fraction (PMF) scores and low positional shift error (PSE) scores (metrics defined in Section 4), implying that most errors arose from misclassification between positive and negative patterns rather than incorrect localization of cleavage sites.

**TABLE 1:** DiCleavePlus cleavage site prediction performance using different pattern sizes.

	Pattern size	Top‐1 accuracy	Macro F1 score	Weighted F1 score	Top‐3 accuracy	PMF	PSE↓
A. Prediction performance on Dataset‐1							
DCP‐AFF	10	0.700 (0.037)	0.378 (0.062)	0.685 (0.049)	0.904 (0.036)	0.700 (0.083)	0.406 (0.091)
DCP‐Concat		0.721 (0.002)	0.414 (0.006)	0.715 (0.005)	0.926 (0.006)	0.735 (0.012)	0.358 (0.018)
DCP‐AFF	12	0.807 (0.017)	0.641 (0.017)	0.803 (0.016)	0.960 (0.008)	0.888 (0.016)	0.168 (0.030)
DCP‐Concat		0.822 (0.010)	0.663 (0.012)	0.820 (0.010)	0.970 (0.004)	0.905 (0.010)	0.144 (0.013)
DCP‐AFF	14	0.861 (0.009)	0.828 (0.011)	0.857 (0.010)	0.979 (0.003)	0.931 (0.004)	0.100 (0.010)
DCP‐Concat		0.864 (0.012)	0.835 (0.018)	0.863 (0.013)	0.980 (0.006)	0.928 (0.014)	0.104 (0.017)
DCP‐AFF	16	0.872 (0.021)	0.864 (0.037)	0.884 (0.020)	0.979 (0.009)	0.924 (0.037)	0.106 (0.039)
DCP‐Concat		0.854 (0.020)	0.837 (0.030)	0.868 (0.018)	0.973 (0.002)	0.895 (0.034)	0.145 (0.035)
DCP‐AFF	18	0.916 (0.008)	0.914 (0.015)	0.926 (0.009)	**0.988 (0.002)**	**0.958 (0.010)**	**0.064 (0.013)**
DCP‐Concat		**0.917 (0.005)**	**0.916 (0.006)**	**0.928 (0.004)**	0.986 (0.003)	0.955 (0.009)	0.068 (0.015)
B. Prediction performance on Dataset‐2							
DCP‐AFF	10	0.553 (0.084)	0.396 (0.061)	0.555 (0.087)	0.832 (0.031)	0.639 (0.099)	0.591 (0.155)
DCP‐Concat		0.573 (0.020)	0.410 (0.013)	0.574 (0.018)	0.832 (0.019)	0.654 (0.024)	0.603 (0.060)
DCP‐AFF	12	0.756 (0.004)	0.646 (0.005)	0.755 (0.005)	0.918 (0.006)	0.836 (0.007)	0.286 (0.034)
DCP‐Concat		0.736 (0.006)	0.630 (0.007)	0.735 (0.007)	0.911 (0.009)	0.816 (0.008)	0.320 (0.026)
DCP‐AFF	14	0.847 (0.008)	0.845 (0.007)	0.846 (0.006)	0.956 (0.003)	0.912 (0.007)	0.167 (0.013)
DCP‐Concat		0.847 (0.029)	0.846 (0.027)	0.847 (0.028)	0.955 (0.009)	0.915 (0.021)	0.170 (0.036)
DCP‐AFF	16	0.892 (0.005)	0.899 (0.005)	0.902 (0.004)	0.969 (0.003)	0.943 (0.005)	0.133 (0.009)
DCP‐Concat		0.892 (0.006)	0.898 (0.006)	0.900 (0.006)	0.966 (0.004)	0.940 (0.006)	0.144 (0.021)
DCP‐AFF	18	**0.915 (0.016)**	**0.919 (0.013)**	**0.922 (0.014)**	**0.976 (0.005)**	**0.956 (0.011)**	**0.088 (0.020)**
DCP‐Concat		0.911 (0.008)	0.915 (0.006)	0.919 (0.007)	0.974 (0.003)	0.955 (0.007)	0.103 (0.015)

*Note:* Average results of fivefold cross‐validation. Numbers in parentheses indicate standard deviation. ↓ indicates the lower value the better performance. Best result is marked in bold.

When the pattern size increased to 18 nt, both models showed further performance improvement, particularly in top‐1 accuracy and F1 scores (macro and weighted). The confusion matrix heatmaps of the best‐performing models on Dataset‐1 are displayed in Figure S3. As shown, cleavage sites positioned near the center of the patterns were predicted accurately, whereas most misclassifications were associated with negative patterns or with positive patterns where cleavage sites were near the pattern boundaries.

The results for Dataset‐2 are summarized in Table 1B. Similar to Dataset‐1, longer pattern sizes consistently led to improved performance. Notably, when pattern sizes were ≥ 14 nt, DiCleavePlus achieved high accuracy on both datasets despite their distinct class distributions, demonstrating the robustness of the proposed architecture. Furthermore, no substantial performance difference was observed between DCP‐AFF and DCP‐Concat, especially when larger pattern sizes were used.

### 2.3 Performance of Positive–Negative Binary Classification

Although DiCleavePlus is designed as a multi‐class classification model, we further evaluated its capability to distinguish positive patterns from negative ones. In this binary evaluation, the original multi‐class output layer was retained. Predictions assigned to Class 0 were regarded as negative, whereas predictions of any nonzero class were considered positive. The binary classification performance of DiCleavePlus is summarized in Table 2. On Dataset‐1, using the empirical 14‐nt pattern size, DCP‐AFF and DCP‐Concat exhibited comparable performance, with DCP‐Concat showing slightly higher sensitivity. When the pattern size increased to 18 nt, both architectures achieved stronger performance in the binary classification task (Table 2A).

**TABLE 2:** DiCleavePlus binary performance using different pattern sizes.

	Pattern size	Accuracy	Specificity	Sensitivity	MCC
A. Prediction performance on Dataset‐1					
DCP‐AFF	10	0.781 (0.030)	0.866 (0.022)	0.665 (0.100)	0.548 (0.063)
DCP‐Concat		0.804 (0.004)	0.850 (0.017)	0.742 (0.029)	0.597 (0.008)
DCP‐AFF	12	0.850 (0.013)	0.866 (0.029)	0.832 (0.029)	0.700 (0.025)
DCP‐Concat		0.860 (0.007)	0.864 (0.021)	0.857 (0.015)	0.721 (0.012)
DCP‐AFF	14	0.892 (0.009)	0.893 (0.009)	0.891 (0.011)	0.784 (0.017)
DCP‐Concat		0.897 (0.007)	0.882 (0.011)	0.912 (0.008)	0.794 (0.014)
DCP‐AFF	16	0.909 (0.006)	0.901 (0.016)	0.915 (0.019)	0.816 (0.012)
DCP‐Concat		0.905 (0.005)	0.909 (0.013)	0.901 (0.018)	0.809 (0.010)
DCP‐AFF	18	0.938 (0.005)	**0.934 (0.009)**	0.940 (0.007)	0.874 (0.009)
DCP‐Concat		**0.940 (0.003)**	0.933 (0.013)	**0.946 (0.009)**	**0.879 (0.006)**
B. Prediction performance on Dataset‐2					
DCP‐AFF	10	0.847 (0.022)	0.341 (0.095)	0.904 (0.029)	0.227 (0.065)
DCP‐Concat		0.856 (0.006)	0.369 (0.039)	0.911 (0.013)	0.262 (0.029)
DCP‐AFF	12	0.897 (0.006)	0.411 (0.065)	0.941 (0.011)	0.345 (0.039)
DCP‐Concat		0.894 (0.007)	0.407 (0.037)	0.939 (0.010)	0.335 (0.021)
DCP‐AFF	14	0.926 (0.005)	0.462 (0.051)	0.962 (0.007)	0.436 (0.021)
DCP‐Concat		0.923 (0.013)	0.507 (0.039)	0.955 (0.014)	0.450 (0.044)
DCP‐AFF	16	0.945 (0.003)	0.482 (0.025)	0.978 (0.003)	0.511 (0.024)
DCP‐Concat		0.946 (0.004)	0.486 (0.054)	0.979 (0.004)	0.521 (0.040)
DCP‐AFF	18	**0.955 (0.007)**	0.511 (0.043)	**0.985 (0.004)**	**0.572 (0.051)**
DCP‐Concat		0.952 (0.003)	**0.522 (0.040)**	0.980 (0.005)	0.553 (0.018)

*Note:* Average results of fivefold cross‐validation. Numbers in parentheses indicate standard deviation. Best result is marked in bold.

In contrast, Dataset‐2 presents an inherently imbalanced binary classification scenario, as negative patterns constitute only approximately 7% of all samples (1624/22,282≈7.29% for 14‐nt patterns and 1624/26,224≈6.19% for 18‐nt patterns). Under this imbalance, for 14‐nt patterns, DCP‐AFF achieved a sensitivity of 0.962, and DCP‐Concat achieved 0.955. However, their specificity scores were substantially lower (0.462 and 0.507, respectively), which consequently led to low Matthews correlation coefficient (MCC) scores of 0.436 and 0.450. Models trained on 18‐nt patterns showed overall improvement, yet their specificity remained limited, resulting in suboptimal MCC scores (Table 2B).

**FIGURE 3:**
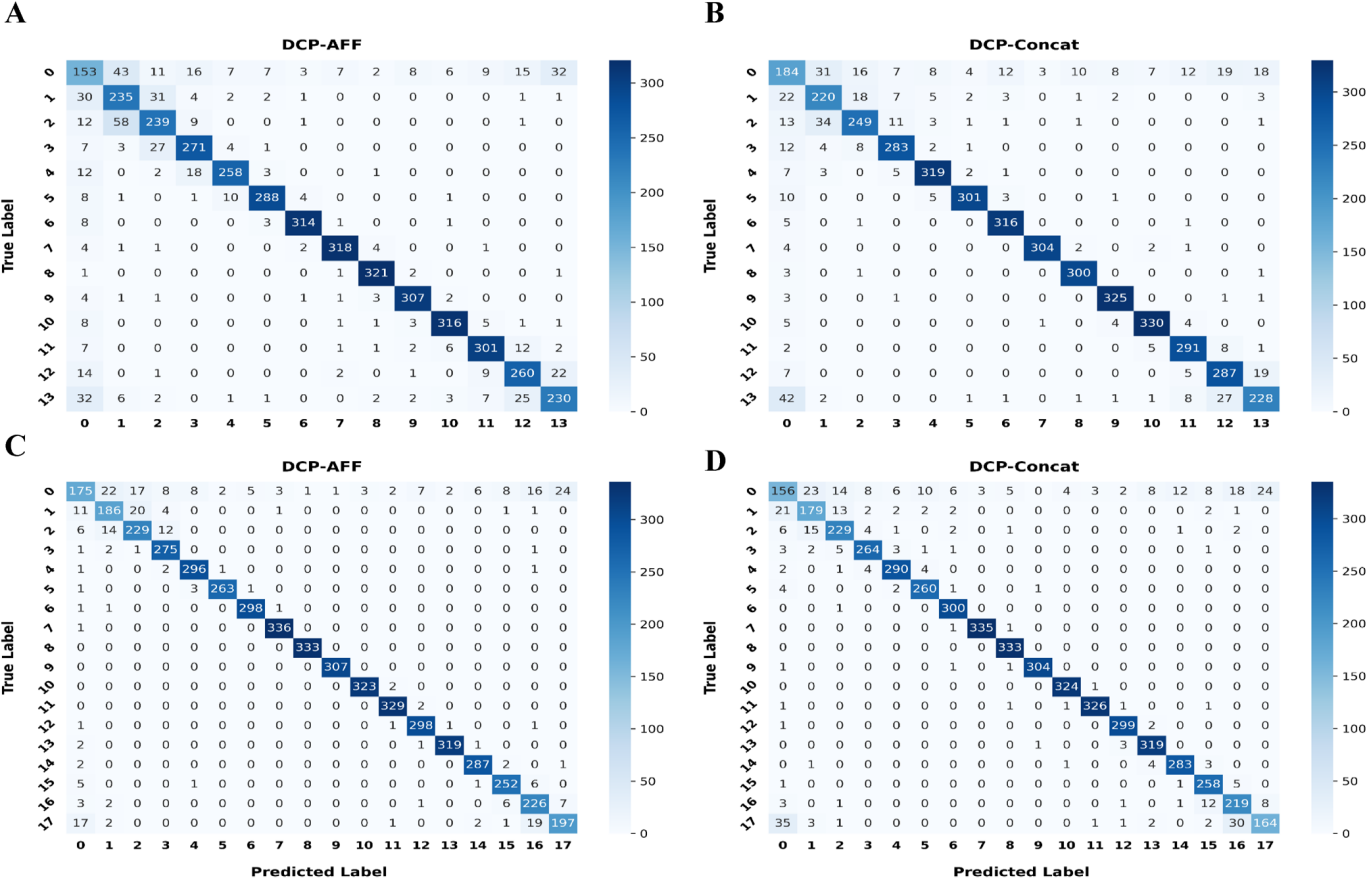
Confusion matrix heatmaps of DiCleavePlus models trained and evaluated on Dataset‐2 using different pattern sizes. (A) DCP‐AFF with 14‐nt patterns; (B) DCP‐Concat with 14‐nt patterns; (C) DCP‐AFF with 18‐nt patterns; and (D) DCP‐Concat with 18‐nt patterns. The *x*‐axis denotes the predicted class; the *y*‐axis denotes the true class. Class 0 corresponds to negative patterns (i.e., patterns that do not contain a cleavage site).

It is important to emphasize that Dataset‐2 is highly imbalanced across classes, and the actual outputs of DiCleavePlus remain intrinsically multi‐class. Therefore, binary evaluation metrics alone do not fully capture the model's predictive behavior. To obtain a more comprehensive understanding, confusion matrix heatmaps of the best‐performing models are presented in Figure 3. All models exhibited similar classification patterns across different pattern sizes. Negative patterns were frequently misclassified as positive patterns with cleavage sites located near boundary intervals. Specifically, mispredictions were concentrated at intervals 1, 2, 13, and 14 in 14‐nt patterns (Figure 3A,B), and at intervals 1, 2, 17, and 18 in 18‐nt patterns (Figure 3C,D). Conversely, positive patterns with cleavage sites at these boundary intervals were often misclassified as negative. Within the positive classes, most errors were also concentrated at these boundary regions rather than in the central intervals. Cleavage sites located near the center of the pattern were predicted with high accuracy, which is consistent with the high PMF and low PSE scores reported in Table 1.

Notably, despite operating in a larger classification space, models trained on 18‐nt patterns outperformed their 14‐nt counterparts in distinguishing between negative patterns and boundary positive patterns. This indicates that longer patterns provide richer contextual cues that enhance discriminative performance, making the 18‐nt configuration the best‐performing model in this study.

### 2.4 Performance Comparison With Ablation Models

The multi‐class classification performance of DiCleavePlus was compared with three ablation models: a multi‐layer perceptron (AM‐MLP), a convolutional neural network (AM‐CNN), and a recurrent neural network (AM‐RNN). As shown in Table 3. DiCleavePlus consistently outperformed all ablation models on both Dataset‐1 and Dataset‐2, under both the empirical pattern size (14 nt) and the extended pattern size (18 nt). Among the ablation models, AM‐RNN achieved the best performance, suggesting that the ability to capture positional dependencies within nucleotide sequences contributes positively to cleavage site prediction.

**TABLE 3:** Performance comparison with DiCleavePlus and its ablation models.

	Pattern size	Top‐1 accuracy	Macro F1 score	Weighted F1 score	Top‐3 accuracy	PMF	PSE↓
A. Prediction performance on Dataset‐1							
DCP‐AFF	14	0.861 (0.009)	0.828 (0.011)	0.857 (0.010)	0.979 (0.003)	0.931 (0.004)	0.100 (0.010)
DCP‐Concat		0.864 (0.012)	0.835 (0.018)	0.863 (0.013)	0.980 (0.006)	0.928 (0.014)	0.104 (0.017)
AM‐MLP		0.512 (0.020)	0.176 (0.015)	0.439 (0.016)	0.714 (0.011)	0.242 (0.024)	1.384 (0.075)
AM‐CNN		0.631 (0.060)	0.420 (0.148)	0.589 (0.088)	0.843 (0.059)	0.558 (0.175)	0.646 (0.342)
AM‐RNN		0.687 (0.020)	0.557 (0.031)	0.674 (0.019)	0.908 (0.009)	0.693 (0.045)	0.395 (0.055)
DCP‐AFF	18	0.916 (0.008)	0.914 (0.015)	0.926 (0.009)	**0.988 (0.002)**	**0.958 (0.010)**	**0.064 (0.013)**
DCP‐Concat		**0.917 (0.005)**	**0.916 (0.006)**	**0.928 (0.004)**	0.986 (0.003)	0.955 (0.009)	0.068 (0.015)
AM‐MLP		0.488 (0.010)	0.168 (0.008)	0.451 (0.004)	0.689 (0.012)	0.216 (0.012)	1.544 (0.102)
AM‐CNN		0.599 (0.079)	0.389 (0.161)	0.587 (0.096)	0.820 (0.079)	0.477 (0.196)	0.852 (0.470)
AM‐RNN		0.682 (0.045)	0.596 (0.088)	0.706 (0.054)	0.901 (0.022)	0.674 (0.083)	0.448 (0.126)
B. Prediction performance on Dataset‐2							
DCP‐AFF	14	0.847 (0.008)	0.845 (0.007)	0.846 (0.006)	0.956 (0.003)	0.912 (0.007)	0.167 (0.013)
DCP‐Concat		0.847 (0.029)	0.846 (0.027)	0.847 (0.028)	0.955 (0.009)	0.915 (0.021)	0.170 (0.036)
AM‐MLP		0.208 (0.005)	0.203 (0.007)	0.202 (0.007)	0.540 (0.008)	0.221 (0.006)	1.798 (0.014)
AM‐CNN		0.456 (0.095)	0.444 (0.094)	0.445 (0.095)	0.798 (0.046)	0.486 (0.103)	0.927 (0.238)
AM‐RNN		0.593 (0.028)	0.592 (0.028)	0.593 (0.027)	0.880 (0.014)	0.668 (0.030)	0.518 (0.059)
DCP‐AFF	18	**0.915 (0.016)**	**0.919 (0.013)**	**0.922 (0.014)**	**0.976 (0.005)**	**0.956 (0.011)**	**0.088 (0.020)**
DCP‐Concat		0.911 (0.008)	0.915 (0.006)	0.919 (0.007)	0.974 (0.003)	0.955 (0.007)	0.103 (0.015)
AM‐MLP		0.200 (0.008)	0.185 (0.020)	0.187 (0.019)	0.529 (0.006)	0.203 (0.012)	1.998 (0.036)
AM‐CNN		0.495 (0.088)	0.502 (0.095)	0.511 (0.097)	0.817 (0.040)	0.522 (0.093)	0.865 (0.162)
AM‐RNN		0.617 (0.124)	0.645 (0.167)	0.654 (0.165)	0.884 (0.047)	0.671 (0.136)	0.546 (0.254)

*Note:* Average results of fivefold cross‐validation. Numbers in parentheses indicate standard deviation. ↓ indicates the lower value the better performance. Best result is marked in bold.

Table 4 presents the binary classification results of DiCleavePlus and the ablation models. The ablation models demonstrated high specificity but low sensitivity on Dataset‐1, indicating a tendency to classify most inputs as negative. In contrast, on Dataset‐2, where negative patterns represent only a small minority, these models achieved high sensitivity but markedly low specificity, revealing their difficulty in distinguishing minority classes under imbalanced distributions. DiCleavePlus, by comparison, maintained a more stable balance between sensitivity and specificity. Notably, it successfully identified approximately 50% of the minority class samples in Dataset‐2, despite the strong class imbalance in the dataset (Table S2).

**TABLE 4:** Binary performance comparison of DiCleavePlus and its ablation models.

	Pattern size	Accuracy	Specificity	Sensitivity	MCC
A. Prediction performance on Dataset‐1					
DCP‐AFF	14	0.892 (0.009)	0.893 (0.009)	0.891 (0.011)	0.784 (0.017)
DCP‐Concat		0.897 (0.007)	0.882 (0.011)	0.912 (0.008)	0.794 (0.014)
AM‐MLP		0.711 (0.011)	0.912 (0.031)	0.517 (0.047)	0.466 (0.016)
AM‐CNN		0.759 (0.043)	0.915 (0.027)	0.607 (0.115)	0.550 (0.064)
AM‐RNN		0.805 (0.008)	0.859 (0.017)	0.751 (0.018)	0.614 (0.016)
DCP‐AFF	18	0.938 (0.005)	0.934 (0.009)	0.940 (0.007)	0.874 (0.009)
DCP‐Concat		**0.940 (0.003)**	0.933 (0.013)	**0.946 (0.009)**	**0.879 (0.006)**
AM‐MLP		0.741 (0.014)	**0.935 (0.024)**	0.583 (0.044)	0.541 (0.014)
AM‐CNN		0.784 (0.031)	0.933 (0.019)	0.662 (0.070)	0.608 (0.043)
AM‐RNN		0.821 (0.025)	0.879 (0.008)	0.773 (0.049)	0.650 (0.042)
B. Prediction performance on Dataset‐2					
DCP‐AFF	14	**0.926 (0.005)**	0.462 (0.051)	0.962 (0.007)	0.436 (0.021)
DCP‐Concat		0.923 (0.013)	**0.507 (0.039)**	0.955 (0.014)	**0.450 (0.044)**
AM‐MLP		0.909 (0.016)	0.129 (0.060)	0.970 (0.019)	0.135 (0.037)
AM‐CNN		0.924 (0.007)	0.173 (0.073)	**0.983 (0.016)**	0.255 (0.034)
AM‐RNN		0.873 (0.006)	0.394 (0.037)	0.911 (0.005)	0.252 (0.034)
DCP‐AFF	18	**0.955 (0.007)**	0.511 (0.043)	**0.985 (0.004)**	**0.572 (0.051)**
DCP‐Concat		0.952 (0.003)	**0.522 (0.040)**	0.980 (0.005)	0.553 (0.018)
AM‐MLP		0.915 (0.015)	0.277 (0.119)	0.957 (0.022)	0.242 (0.048)
AM‐CNN		0.932 (0.007)	0.268 (0.077)	0.976 (0.011)	0.304 (0.046)
AM‐RNN		0.909 (0.014)	0.369 (0.084)	0.944 (0.018)	0.288 (0.047)

*Note:* Average results of fivefold cross‐validation. Numbers in parentheses indicate standard deviation. Best result is marked in bold.

These results demonstrate the importance of Transformer‐based feature extractors used in DiCleavePlus. The superior performance, particularly in detecting minority classes, indicates that attention‐based mechanisms enable more effective modeling of both local and long‐range sequence‐structure dependencies compared with simpler MLP‐, CNN‐, or RNN‐based architectures.

### 2.5 Performance Comparison With Other Models

We compared DiCleavePlus with two state‐of‐the‐art models: ReCGBM (Liu et al. 2021) and DiCleave (Mu et al. 2024). The results of multi‐class classification and binary classification are summarized in Tables 5 and 6, respectively. Across both tasks, DiCleavePlus consistently outperformed the competing models. As a deep learning model, DiCleave achieved better performance than ReCGBM on Dataset‐1 and demonstrated comparable performance on Dataset‐2. However, compared with the three ablation models (Tables 3 and 4), DiCleave outperformed only the MLP‐based ablation model, whereas it fell behind the CNN‐ and RNN‐based ablation models.

**TABLE 5:** Performance comparison with DiCleavePlus and other models.

	Pattern size	Top‐1 accuracy	Macro F1 score	Weighted F1 score	Top‐3 accuracy	PMF	PSE↓
A. Prediction performance on Dataset‐1							
DCP‐AFF	14	0.861 (0.009)	0.828 (0.011)	0.857 (0.010)	0.979 (0.003)	**0.931 (0.004)**	**0.100 (0.010)**
DCP‐Concat		**0.864 (0.012)**	**0.835 (0.018)**	**0.863 (0.013)**	**0.980 (0.006)**	0.928 (0.014)	0.104 (0.017)
DiCleave		0.515 (0.017)	0.192 (0.030)	0.452 (0.016)	0.723 (0.030)	0.243 (0.046)	1.507 (0.310)
ReCGBM		0.499 (0.007)	0.129 (0.006)	0.380 (0.008)	NA	0.294 (0.021)	1.948 (0.083)
B. Prediction performance on Dataset‐2							
DCP‐AFF	14	**0.847 (0.008)**	0.845 (0.007)	0.846 (0.006)	**0.956 (0.003)**	0.912 (0.007)	**0.167 (0.013)**
DCP‐Concat		0.847 (0.029)	**0.846 (0.027)**	**0.847 (0.028)**	0.955 (0.009)	**0.915 (0.021)**	0.170 (0.036)
DiCleave		0.222 (0.028)	0.210 (0.039)	0.209 (0.039)	0.576 (0.054)	0.242 (0.027)	1.750 (0.333)
ReCGBM		0.224 (0.002)	0.224 (0.002)	0.224 (0.002)	NA	0.244 (0.005)	2.766 (0.051)

*Note:* Average results of fivefold cross‐validation. Numbers in parentheses indicate standard deviation. ↓ indicates the lower value the better performance. Best result is marked in bold.

**TABLE 6:** Binary performance comparison of DiCleavePlus and other models.

	Pattern size	Accuracy	Specificity	Sensitivity	MCC
A. Prediction performance on Dataset‐1					
DCP‐AFF	14	0.892 (0.009)	0.893 (0.009)	0.891 (0.011)	0.784 (0.017)
DCP‐Concat		**0.897 (0.007)**	0.882 (0.011)	**0.912 (0.008)**	**0.794 (0.014)**
DiCleave		0.724 (0.018)	0.913 (0.023)	0.541 (0.054)	0.488 (0.021)
ReCGBM		0.565 (0.008)	**0.959 (0.003)**	0.183 (0.006)	0.224 (0.012)
B. Prediction performance on Dataset‐2					
DCP‐AFF	14	**0.926 (0.005)**	0.462 (0.051)	**0.962 (0.007)**	0.436 (0.021)
DCP‐Concat		0.923 (0.013)	**0.507 (0.039)**	0.955 (0.014)	**0.450 (0.044)**
DiCleave		0.878 (0.017)	0.165 (0.039)	0.934 (0.017)	0.101 (0.048)
ReCGBM		0.873 (0.006)	0.189 (0.032)	0.926 (0.007)	0.109 (0.026)

*Note:* Average results of fivefold cross‐validation. Numbers in parentheses indicate standard deviation. Best result is marked in bold.

The limited performance of DiCleave can be attributed to several factors. First, DiCleave employs one‐hot encoding to represent the input sequence. This encoding scheme is insufficient to capture nuanced differences between highly similar patterns, especially in this study, where most Cleavage Patterns, particularly the positive ones, substantially overlap. Consequently, the model may fail to discriminate between patterns that differ only in subtle yet functionally important positions. Second, DiCleave processes Cleavage Patterns and full‐length pre‐miRNA sequences separately. The features of pre‐miRNA sequences are derived from a pretrained autoencoder, and the model does not jointly process the Cleavage Pattern alongside its original pre‐miRNA context. The isolated processing hinders the model from leveraging the contextual relationship between a pattern and its structural environment within the pre‐miRNA. Third, the CNN‐based feature extractor in DiCleave does not incorporate positional encoding in its input representation. Without explicit positional information, the model may struggle to learn the spatial arrangement of nucleotides within Cleavage Patterns, thereby limiting its representational capacity, especially for tasks that require precise localization of cleavage sites.

In contrast, DiCleavePlus addresses these limitations by integrating embedding‐based representations, joint processing of Cleavage Patters and full‐length pre‐miRNA sequences, and the use of Transformer encoders with positional encoding. As a result, DiCleavePlus achieves superior performance in both multi‐class and binary prediction tasks and better captures positional and contextual dependencies critical for Dicer cleavage site prediction.

## 3 Discussion

Precise Dicer cleavage is essential for the biogenesis of mature miRNAs. Extensive in vivo and in vitro studies have been conducted to elucidate the underlying principles of Dicer processing. The studies have demonstrated that Dicer does not cleave randomly but instead follows defined rules, such as the 3′ counting rules (MacRae et al. 2006, 2007) and the 5′ counting rules (Park et al. 2011). Subsequent findings further revealed that both the secondary structure of pre‐miRNAs and the presence of specific sequence motifs play crucial roles in determining cleavage sites (Gu et al. 2012; Nguyen et al. 2022; Le et al. 2024). In addition to its role in miRNA maturation, Dicer also participates in the production of small interfering RNAs (siRNAs) by cleaving double‐stranded RNAs (Elbashir et al. 2001; Bernstein et al. 2001; Ketting et al. 2001; Knight and Bass 2001). Given that siRNAs are considered a promising therapeutic modality (Weng et al. 2019; Sardh et al. 2019; Agarwal et al. 2020; Hu et al. 2020), a comprehensive understanding of Dicer cleavage specificity is of both biological and clinical importance. Although previous studies have provided substantial insights into the mechanism governing Dicer cleavage, experimental approaches remain labor‐intensive, time‐consuming, and limited in scale. Consequently, the development of accurate computational models for Dicer cleavage site prediction is indispensable and will significantly facilitate research on miRNA biogenesis, discovery of potential miRNA products, and the rational design of siRNA precursors.

Several computational approaches have been proposed to predict human Dicer cleavage sites. PHDcleav, LBSizeCleav, and ReCGBM are Cleavage Pattern‐based machine learning models. Due to their limited use of contextual information, Cleavage Patterns derived from the 5′ and 3′ arms of the same pre‐miRNA are considered independent entities in these models. In contrast, DiCleave integrates full‐length pre‐miRNA sequences and therefore requires no additional prior knowledge of the input Cleavage Patterns, making it a more practical tool. However, DiCleave only recognizes cleavage sites located at the central position of the patterns, essentially reducing the task to binary classification and failing to fully utilize positional diversity within Cleavage Patterns.

In this study, we proposed DiCleavePlus to address these limitations. By treating each interval within a Cleavage Pattern as a unique class, we reformulated the task as a multi‐class classification problem. In this new setting, Dicer cleavage sites can occur at any interval within the patterns, rather than being restricted to the central position. This design not only allows for a more comprehensive representation of Dicer cleavage sites but also effectively increases the number of training samples. For example, when using a 14‐nt pattern, a single cleavage site yields 13 distinct positive patterns, whereas conventional Cleavage Patterns generate only one. In this sense, DiCleavePlus introduces an implicit form of data augmentation, mitigating the limitation of insufficient training data for deep learning architectures. We represented pre‐miRNA sequences and dot‐bracket secondary structures using 3‐mer tokens with 32‐dimensional embedding and employed Transformer encoders to extract features from both Cleavage Patterns and full‐length pre‐miRNA sequences. To integrate the information from these two sources, an AFF module was implemented.

Our experimental results demonstrate that DiCleavePlus achieved promising predictive performance. Its accuracy improved with longer pattern sizes, which is consistent with observations reported in PHDcleav and LBSizeCleav. While 14‐nt patterns yielded satisfactory results, the best performance was achieved using 18‐nt patterns, indicating that extended patterns provide additional structural and sequence‐based contextual information that facilitates precise cleavage site localization. DiCleavePlus achieved approximately 91% top‐1 accuracy and 98% top‐3 accuracy with 18‐nt patterns, suggesting that correct predictions were almost always ranked among the top candidates. This high level of accuracy underscores the robustness and practical applicability of the model.

The main limitation of DiCleavePlus lies in its difficulty in distinguishing negative patterns from positive patterns in which cleavage sites are located near the boundary intervals. In contrast, cleavage sites located near the center of the patterns were predicted with high accuracy, suggesting that the flanking regions surrounding the cleavage site play an essential role in determining the cleavage site. Hence, boundary‐localized cleavage sites lack complete upstream or downstream flanking region information, which likely contributes to their frequent misclassification (Figures 3 and S3). This phenomenon suggests that the current pattern‐based representation still has inherent constraints in capturing the full structural landscape required for cleavage site recognition.

Another source of potential limitation arises from the RNA secondary structures used in this study. All pre‐miRNA secondary structures were predicted by RNAfold (Hofacker 2003), which does not allow pseudoknot structures. Since substrate structures have been reported to influence Dicer processing (MacRae et al. 2007; Feng et al. 2012), pseudoknot structures may likewise play an important role in determining Dicer cleavage sites. In cases where the 5′ and 3′ cleavage sites are spatially adjacent, pseudoknot‐like interactions could impose local structural constraints that guide Dicer activity. The absence of such structural information in the current dataset may therefore limit the representational capacity of DiCleavePlus to fully capture these context‐dependent Cleavage Patterns.

We also observed that there was no substantial difference in performance between the two feature fusion strategies, DCP‐AFF and DCP‐Concat. Further analysis of attention weights revealed that the AFF module exhibited a strong bias toward pattern features, with attention contributions ranging from 0.95 to 0.99, suggesting that the current architecture may not fully capture or utilize information from full‐length pre‐miRNA sequences. Future improvements could involve more effective sequence embedding strategies, such as multi‐view multi‐scale encoding (He et al. 2021; Zhang et al. 2024), to enhance the contribution of sequence context. Additionally, the AFF module adopted in this work corresponds to the basic implementation described by Dai et al. (2021). Their iterative attentional feature fusion (iAFF) architecture, which repeatedly computes feature contributions by stacking multiple multi‐scale channel attention blocks, may enhance the model's capacity to integrate heterogeneous features. Expanding DiCleavePlus to incorporate such advanced feature fusion strategies could further improve prediction accuracy and robustness.

## 4 Experimental Procedures

### 4.1 Data Preparation

#### 4.1.1 Data Collection

A total of 956 human pre‐miRNAs that explicitly contain two mature miRNAs were retrieved from the miRBase database (Release 22.1, https://www.mirbase.org/download/). As illustrated in Figure S1, the positions of Dicer cleavage sites can be reliably inferred from the annotated mature miRNA regions within these pre‐miRNAs. To reduce sequence redundancy, CD‐HIT‐EST (Li and Godzik 2006; Fu et al. 2012) was applied using an 80% similarity threshold (see Supporting Information: File S1 for a detailed discussion of this choice). After filtering, 812 pre‐miRNA sequences were retained, each containing two Dicer cleavage sites.

For each pre‐miRNA sequence, all possible Cleavage Patterns were generated in a sliding window manner. Specifically, if the pre‐miRNA has a length of L and the pattern size is denoted by s, then it yields a total of L−s+1 Cleavage Patterns. Patterns that inadvertently encompassed two cleavage sites, which occurred when the 5′ and 3′ cleavage sites were in close proximity, were removed to ensure that each pattern contained at most one cleavage site. If a pattern contained a cleavage site at its *k*‐th interval, it was assigned to Class *k*; otherwise, it was labeled as Class 0. In our datasets, each sample entry is a Cleavage Pattern, which is associated with other information, including the corresponding pattern secondary structure, the full‐length pre‐miRNA sequence from which it originates, and the secondary structure of that pre‐miRNA. The detailed configuration of dataset is presented in Table S1.

To construct Dataset‐1, all positive patterns were retained, and 20,000 negative patterns were randomly sampled. Because the negative class substantially outnumbered each positive class in Dataset‐1, a class‐balanced dataset, Dataset‐2, was constructed by randomly selecting two negative patterns per pre‐miRNA. To investigate the influence of pattern size on model performance, we set $s\in \left\{10,12,14,16,18\right\}$, resulting in five variants of Dataset‐1 and Dataset‐2 using different pattern sizes s. Table S2 summarizes the number of patterns per class in each variant of Dataset‐1 and Dataset‐2. A visual overview of the data collection and processing pipeline is provided in Figure S2.

#### 4.1.2 Cross‐Validation Strategy

A fivefold cross‐validation strategy was employed to evaluate the performance of DiCleavePlus. The dataset was randomly partitioned into five equal subsets at the sample (Cleavage Pattern) level, meaning that each individual Cleavage Pattern was treated as an independent unit when assigning samples to folds. Each Cleavage Pattern was characterized by four components: the pattern sequence, the corresponding pattern secondary structure, the full‐length pre‐miRNA sequence from which it originates, and the secondary structure of that pre‐miRNA. To reduce redundancy and limit potential information leakage, redundant pre‐miRNA sequences were removed using CD‐HIT‐EST with an 80% similarity threshold (Supporting Information: File S1 and Figure S7). In addition, DiCleavePlus was designed to incorporate all four components as its inputs, ensuring that no two input samples are identical. Nevertheless, we acknowledge that the sliding window‐based pattern generation inherently produces patterns with overlapping sequence and structural context. Consequently, a shifted window derived from the same pre‐miRNA may appear in a different fold, particularly for negative patterns that share the same class label. Although this residual dependence does not compromise the overall validity of the evaluation, it represents a limitation of pattern‐level cross‐validation and should be considered when interpreting the results.

In each fold, one subset was held out as the test set, while the remaining four subsets were further split into a training set and a validation set, with 90% of the data allocated for training and 10% for validation to monitor the training process. This process was repeated five times, with each subset served once as the test set, and the final performance was reported as the average across the five folds.

### 4.2 Data Processing

#### 4.2.1 Pre‐miRNA Secondary Structure

The secondary structures of pre‐miRNAs were predicted using RNAfold (Hofacker 2003), and represented in dot‐bracket notation. Because RNAfold predicts pseudoknot‐free structures, only three types of symbols are used in the output: an opening bracket “(” and a closing bracket “)” to represent base‐paired nucleotides, and a dot “.” to indicate unpaired nucleotides. An example of this representation is shown in Figure S4A (green part).

#### 4.2.2 K‐Mer Encoding

We utilized 3‐mer tokens to represent the pre‐miRNA sequences and the corresponding secondary structures. For each sequence, overlapping 3‐mer tokens were generated. Since the last two elements of a sequence cannot form complete 3‐mer, placeholder symbols were appended to ensure that the total number of 3‐mer tokens matched the original sequence length. For instance, the penultimate and the final nucleotides of a pre‐miRNA sequence are represented as “NN<PH>” and “N<PH><PH>,” respectively, where “N” denotes any nucleotide, and “<PH>” indicates a placeholder.

Based on this scheme, a vocabulary of 84 unique 3‐mer tokens was constructed for nucleotide sequences, while the dot‐bracket secondary structures formed a vocabulary of 39 unique 3‐mer tokens. Each 3‐mer token was subsequently embedded into a 32‐dimensional continuous vector space and used as the input representation for both sequence and secondary structure (Figure S4A).

#### 4.2.3 Input Features of DiCleavePlus

To ensure consistent input length across samples, the embedding vectors of pre‐miRNA sequences and their corresponding secondary structure were padded to a fixed length of 200. The sequence embeddings were then concatenated with the corresponding secondary structure embeddings to form the full sequence feature tensors. To preserve positional information essential for sequential modeling, sinusoid positional encodings (Vaswani et al. 2017) were added to the combined embeddings.

A similar procedure was applied to the Cleavage Pattern inputs. The embeddings of each Cleavage Pattern were concatenated with their respective secondary structure embeddings, followed by the addition of sinusoid positional encodings. These processed Cleavage Pattern features, together with the pre‐miRNA sequence features, served as the dual input streams to DiCleavePlus (Figure S4B).

### 4.3 AFF

AFF from Dai et al. (2021) was utilized to integrate Cleavage Pattern features and pre‐miRNA sequence features processed by the Encoder Unit. The core component of AFF, termed the multi‐scale channel attention block, computes global and local contexts of input features separately. These contexts are subsequently aggregated and passed through a sigmoid function to calculate the attention weight. AFF then fuses the two weighted input features by summing them.

$$Z=M\left(X⊕Y\right)⊗X+\left(1-M\left(X⊕Y\right)\right)⊗Y$$

where Z denotes the output fused feature; X and Y indicate two input features; and $M\left(\right)$ denotes a multi‐scale channel attention block. The output of $M\left(\right)$ is a tensor with values in the range [0, 1], which is used as the weight for X; $1-M\left(\right)$ is used as the weight of Y, which is complementary to the weight of X; ⊕ denotes element‐wise addition; and ⨂ denotes element‐wise multiplication.

The AFF module implemented in DiCleavePlus is illustrated in Figure S5.

### 4.4 Comparison With Ablation Models

To evaluate the contribution of Transformer‐based feature extractors within DiCleavePlus, we constructed three ablation models by replacing the Encoder Unit with alternative architectures: an MLP, a CNN, and an RNN. For the CNN‐based ablation model, sinusoidal positional encodings were additionally incorporated to enable sensitivity to positional dependencies within the input sequences, compensating for the inherent locality of convolutional operations. All ablation models were trained and evaluated under the same experimental settings as DiCleavePlus, using both 14‐nt and 18‐nt datasets to ensure a fair comparison.

### 4.5 Comparison With Other Models

Although DiCleave employs a multi‐class output layer (Mu et al. 2024), its prediction task is essentially binary, as it only predicts whether a Dicer cleavage site is located at the center of a given Cleavage Pattern. To ensure a fair comparison with DiCleavePlus, we modified DiCleave by replacing its output layer with that of our proposed model, thereby adapting it to the multi‐class setting of our study.

Similarly, we adapted ReCGBM, another state‐of‐the‐art architecture, by modifying its source code to enable multi‐class classification, as the original model was designed exclusively for binary predictions.

Since DiCleave and ReCGBM were originally designed to process Cleavage Patterns of 14 nt, for comparative evaluation, we retrained and evaluated these models using the same 14‐nt datasets employed in this study. All training procedures and hyperparameter configurations for DiCleave and ReCGBM were preserved as in their original implementations, ensuring the validity and fairness of the performance comparison with DiCleavePlus.

### 4.6 Performance Metrics

#### 4.6.1 Metrics to Assess Multi‐Classification Performance

DiCleavePlus is designed as a multi‐class classification model. Accordingly, its performance was evaluated using top‐1 accuracy, macro F1 score, weighted F1 score, PMF, PSE, and top‐3 accuracy.

Top‐1 accuracy was defined as the proportion of patterns for which the predicted class exactly matched the labeled class:

$$\mathrm{Top}1\;\text{accuracy}=\frac{\sum 1\left(\text{pred}=\mathrm{lbl}\right)}{N}$$

where N indicates the total number of patterns; pred denotes predicted cleavage site position; lbl denotes actual cleavage site position; and $1\left(\text{pred}=\mathrm{lbl}\right)$ is an indicator variable, which is set to 1 when the prediction matches the label.

To account for performance across all classes, we calculated the macro F1 score:

$$\text{Macro}\;F1=\frac{1}{N_{C}}\sum F1\;{\text{score}}_{c}$$

$$F1\;{\text{score}}_{c}=\frac{2\times {\mathrm{TP}}_{c}}{2\times {\mathrm{TP}}_{c}+\left({\mathrm{FP}}_{c}+{\mathrm{FN}}_{c}\right)}$$

where $N_{C}$ indicates total number of classes; $F1\;{\text{score}}_{c}$ indicates the F1 score per‐class; and ${\mathrm{TP}}_{c}$, ${\mathrm{FP}}_{c}$, and ${\mathrm{FN}}_{c}$ denote the numbers of true positives, false positives, and false negatives for each class, respectively.

The weighted F1 score was also computed to account for class imbalance:

$$\text{Weighted}\;F1=\sum w_{c}\times F1\;{\text{score}}_{c}$$

$$w_{c}=\frac{\sum 1\left(c_{i}\in c\right)}{N}$$

where $w_{c}$ is defined as the proportion of the number of patterns in class c to the total number of patterns; $1\left(c_{i}\in c\right)$ is an indicator variable, which is set to 1 when the class of pattern i belongs to class c.

In this study, each class represents a specific interval within a Cleavage Pattern corresponding to a potential Dicer cleavage site. To more precisely assess the positional accuracy of cleavage site prediction, we employed the PMF and PSE (Bell and Hendrix 2022). Since Class 0 represents negative patterns with no cleavage site, PSE is undefined for samples where the true or predicted class is 0. Therefore, PMF and PSE were calculated only for true positives:

$$\mathrm{PMF}=\frac{\sum 1\left(\text{pred}=\mathrm{lbl}\right)}{N_{\mathrm{TP}}}\;\text{pred}\neq 0,\mathrm{lbl}\neq 0$$

$$\mathrm{PSE}=\frac{\sum |\text{pred}-\mathrm{lbl}|}{N_{\mathrm{TP}}}\;\text{pred}\neq 0,\mathrm{lbl}\neq 0$$

where $N_{\mathrm{TP}}$ indicates the total number of true positive patterns; pred denotes predicted cleavage site position; lbl denotes actual cleavage site position; and $1\left(\text{pred}=\mathrm{lbl}\right)$ is an indicator variable, which is set to 1 when the prediction matches the label. PMF accesses the proportion of predictions in which the predicted class exactly matches the labeled class, while PSE quantifies the positional shift between predicted and labeled class.

Finally, to assess the practical utility of the model, we also reported the top‐3 accuracy, which measures the proportion of samples for which the correct class is ranked among the three most probable model predictions. This metric is especially relevant in experimental scenarios, where a small set of high‐confidence candidate sites is often sufficient for further validation.

#### 4.6.2 Metrics to Assess Binary Classification Performance

In addition to multi‐class evaluation, we further assessed the ability of DiCleavePlus to distinguish positive patterns from negative ones. For this purpose, conventional binary classification metrics were employed, including accuracy (Acc), specificity (Spe), sensitivity (Sen), and MCC.

Accuracy was defined as the proportion of correctly classified samples:

$$\mathrm{Acc}=\frac{\mathrm{TP}+\mathrm{TN}}{\mathrm{TP}+\mathrm{TN}+\mathrm{FP}+\mathrm{FN}}$$

Specificity measures the proportion of true negative samples that were correctly classified:

$$\mathrm{Spe}=\frac{\mathrm{TN}}{\mathrm{TN}+\mathrm{FP}}$$

Sensitivity quantifies the proportion of true positive samples that were correctly identified:

$$\mathrm{Sen}=\frac{\mathrm{TP}}{\mathrm{TP}+\mathrm{FN}}$$

The MCC was used as a balanced metric to evaluate the overall quality of binary classification, particularly under class‐imbalanced conditions:

$$\mathrm{MCC}=\frac{\mathrm{TP}\times \mathrm{TN}-\mathrm{FP}\times \mathrm{FN}}{\sqrt{\left(\mathrm{TP}+\mathrm{FP}\right)\times \left(\mathrm{TP}+\mathrm{FN}\right)\times \left(\mathrm{TN}+\mathrm{FP}\right)\times \left(\mathrm{TN}+\mathrm{FN}\right)}}$$

where TP, TN, FP, and FN denote the numbers of true positives, true negatives, false positives, and false negatives, respectively.

### 4.7 Details of Training Procedure

A fivefold cross‐validation strategy was employed to evaluate model performance. The dataset was randomly partitioned into five equal subsets. In each fold, one subset was held out as the test set, while the remaining four subsets were used for model development. Among these four subsets, 10% of the samples were randomly selected to form the validation set, and the remaining 90% were used for training. This process was repeated five times to ensure that each subset served once as the test set.

The model was trained with a mini‐batch size of 128, which was chosen as a compromise between computational efficiency and model stability. Optimization was performed using the AdamW optimizer (Loshchilov and Hutter 2017), with an initial learning rate of 0.001 and weight decay set to 1e−5.

The maximum number of training epochs was set to 100. At the end of each epoch, both the training and validation sets were shuffled prior to the next epoch to avoid learning biases from fixed sample orders. A learning rate scheduler was applied to reduce the learning rate by a factor of 0.5 when the validation loss failed to decrease. In addition, an early‐stopping mechanism based on validation loss was employed to terminate training when no further improvement was observed.

For each fold, the final checkpoint of the trained model and up to three checkpoints corresponding to the highest validation performance were saved for subsequent analysis. The checkpoint with the best performance on the test set was selected as the representative model for that fold.

All experiments were conducted on an NVIDIA H100 Tensor Core GPU with 64 GB of memory. The computational time required for training is summarized in Table S3.

## Author Contributions

**Lixuan Mu:** conceptualization, investigation, data curation, methodology, software, formal analysis, visualization, writing – original draft, writing – review and editing. **Tatsuya Akutsu:** conceptualization, funding acquisition, project administration, supervision, writing – review and editing.

## Funding

This work has been supported by the Japan Society for the Promotion of Science (22H00532 to T.A., 22K19830 to T.A.).

## Ethics Statement

The authors have nothing to report.

## Conflicts of Interest

The authors declare no conflicts of interest.

## Supporting information


**Figure S1:** Linear representation of human pre‐miRNA hsa‐let‐7a. The blue‐shaded regions correspond to two mature miRNAs, hsa‐let‐7a‐5p, and hsa‐let‐7a‐3p. The position of Dicer cleavage sites (red triangles) and Drosha cleavage sites (blue triangles) is inferred from these mature miRNAs. Red frames highlight 14‐nt Cleavage Patterns in which the Dicer cleavage site is located at the central interval.
**Figure S2:** Workflow for dataset construction.
**Figure S3:** Confusion matrix heatmaps of DiCleavePlus models trained and evaluated on Dataset‐1 with different pattern sizes. (A) DCP‐AFF with 14‐nt patterns; (B) DCP‐Concat with 14‐nt patterns; (C) DCP‐AFF with 18‐nt patterns; and (D) DCP‐Concat with 18‐nt patterns. The *x*‐axis denotes the predicted class; the *y*‐axis denotes the true class. Class 0 corresponds to negative patterns (i.e., patterns that do not contain a cleavage site).
**Figure S4:** Data processing workflow of DiCleavePlus. (A) Pre‐miRNA sequences (blue blocks) and their corresponding secondary structures (green blocks) are tokenized into 3‐mers and encoded using 32‐dimensional embeddings. (B) The sequence embeddings are concatenated with the secondary structure embeddings to generate the input features of DiCleavePlus.
**Figure S5:** Architecture of the attentional feature fusion (AFF) block used in this study. The orange module represents the multi‐scale channel attention block. The symbol ⊕ denotes element‐wise addition, and ⊗ indicates element‐wise multiplication. The dashed line indicates the computation of complementary weight (1−w).
**Figure S6:** Heatmap of pairwise sequence similarity matrix among the original 956 pre‐miRNAs. (A) Heatmap of pairwise alignment score matrix computed using the BLASTN scoring scheme. (B) Heatmap of pairwise similarity score matrix.
**Figure S7:** Heatmap of pairwise sequence similarity matrix among pre‐miRNAs after applying the 80% CD‐HIT‐EST threshold. (A) Heatmap of pairwise alignment score matrix computed using the BLASTN scoring scheme. (B) Heatmap of pairwise similarity score matrix.
**Figure S8:** Number of pre‐miRNA clusters under different sequence similarity thresholds. The *x*‐axis represents the similarity threshold used for clustering, and the *y*‐axis represents the corresponding number of pre‐miRNA clusters.

**Data S1:** Figure Legends.

**Table S1:** Dataset configuration.

**Table S2:** The number of patterns per class in different pattern length datasets.

**Table S3:** Average training time of DiCleavePlus (minutes).

**Table S4:** DiCleavePlus cleavage site prediction performance using different pre‐miRNA sequence cutoff thresholds.

**Table S5:** DiCleavePlus binary performance using different pre‐miRNA sequence cutoff thresholds.

**File S1:** Discussion about similarity cutoff threshold.

## Data Availability

The raw data, including pre‐miRNA hairpin sequences and mature miRNA sequences, were obtained from the miRBase database (Release 22.1, https://www.mirbase.org/download/). The source code of this study is available at https://github.com/MGuard0303/DiCleavePlus. Datasets used in this study are available at https://github.com/MGuard0303/DiCleavePlus/releases/tag/data.

## References

[gtc70074-bib-0001] Agarwal, S. , A. R. Simon , V. Goel , et al. 2020. “Pharmacokinetics and Pharmacodynamics of the Small Interfering Ribonucleic Acid, Givosiran, in Patients With Acute Hepatic Porphyria.” Clinical Pharmacology & Therapeutics 108, no. 1: 63–72. 31994716 10.1002/cpt.1802

[gtc70074-bib-0002] Ahmed, F. , R. Kaundal , and G. P. Raghava . 2013. “PHDcleav: A SVM Based Method for Predicting Human Dicer Cleavage Sites Using Sequence and Secondary Structure of miRNA Precursors.” BMC Bioinformatics 14, no. Suppl 14: S9. 10.1186/1471-2105-14-S14-S9 PMC3851333 24267009

[gtc70074-bib-0003] Ali Syeda, Z. , S. S. S. Langden , C. Munkhzul , M. Lee , and S. J. Song . 2020. “Regulatory Mechanism of MicroRNA Expression in Cancer.” International Journal of Molecular Sciences 21, no. 5: 1723. 32138313 10.3390/ijms21051723 PMC7084905

[gtc70074-bib-0004] Bao, Y. , M. Hayashida , and T. Akutsu . 2016. “LBSizeCleav: Improved Support Vector Machine (SVM)‐Based Prediction of Dicer Cleavage Sites Using Loop/Bulge Length.” BMC Bioinformatics 17, no. 1: 487. 27887571 10.1186/s12859-016-1353-6 PMC5124314

[gtc70074-bib-0005] Bell, J. , and D. A. Hendrix . 2022. “Predicting Drosha and Dicer Cleavage Sites With DeepMirCut.” Frontiers in Molecular Biosciences 8: 799056. 35141278 10.3389/fmolb.2021.799056 PMC8819831

[gtc70074-bib-0006] Bernstein, E. , A. A. Caudy , S. M. Hammond , and G. J. Hannon . 2001. “Role for a Bidentate Ribonuclease in the Initiation Step of RNA Interference.” Nature 409, no. 6818: 363–366. 11201747 10.1038/35053110

[gtc70074-bib-0007] Calin, G. A. , C. D. Dumitru , M. Shimizu , et al. 2002. “Frequent Deletions and Down‐Regulation of Micro‐RNA Genes miR15 and miR16 at 13q14 in Chronic Lymphocytic Leukemia.” Proceedings of the National Academy of Sciences of the United States of America 99, no. 24: 15524–15529. 12434020 10.1073/pnas.242606799 PMC137750

[gtc70074-bib-0008] Care, A. , D. Catalucci , F. Felicetti , et al. 2007. “MicroRNA‐133 Controls Cardiac Hypertrophy.” Nature Medicine 13, no. 5: 613–618. 10.1038/nm1582 17468766

[gtc70074-bib-0009] Dai, Y. , F. Gieseke , S. Oehmcke , Y. Wu , and K. Barnard . 2021. “Attentional Feature Fusion.” In Proceedings of the IEEE/CVF Winter Conference on Applications of Computer Vision, 3560–3569. IEEE.

[gtc70074-bib-0010] Elbashir, S. M. , J. Harborth , W. Lendeckel , A. Yalcin , K. Weber , and T. Tuschl . 2001. “Duplexes of 21‐Nucleotide RNAs Mediate RNA Interference in Cultured Mammalian Cells.” Nature 411, no. 6836: 494–498. 11373684 10.1038/35078107

[gtc70074-bib-0011] Feng, Y. , X. Zhang , P. Graves , and Y. A. N. Zeng . 2012. “A Comprehensive Analysis of Precursor microRNA Cleavage by Human Dicer.” RNA 18, no. 11: 2083–2092. 22984192 10.1261/rna.033688.112 PMC3479397

[gtc70074-bib-0012] Fu, L. , B. Niu , Z. Zhu , S. Wu , and W. Li . 2012. “CD‐HIT: Accelerated for Clustering the Next‐Generation Sequencing Data.” Bioinformatics 28, no. 23: 3150–3152. 23060610 10.1093/bioinformatics/bts565 PMC3516142

[gtc70074-bib-0013] Griffiths‐Jones, S. , H. K. Saini , S. Van Dongen , and A. J. Enright . 2007. “miRBase: Tools for microRNA Genomics.” Nucleic Acids Research 36, no. Suppl_1: D154–D158. 17991681 10.1093/nar/gkm952 PMC2238936

[gtc70074-bib-0014] Gu, S. , L. Jin , Y. Zhang , et al. 2012. “The Loop Position of shRNAs and Pre‐miRNAs Is Critical for the Accuracy of Dicer Processing In Vivo.” Cell 151, no. 4: 900–911. 23141545 10.1016/j.cell.2012.09.042 PMC3499986

[gtc70074-bib-0015] Ha, M. , and V. N. Kim . 2014. “Regulation of MicroRNA Biogenesis.” Nature Reviews Molecular Cell Biology 15, no. 8: 509–524. 25027649 10.1038/nrm3838

[gtc70074-bib-0016] Hammond, S. M. , E. Bernstein , D. Beach , and G. J. Hannon . 2000. “An RNA‐Directed Nuclease Mediates Post‐Transcriptional Gene Silencing in Drosophila Cells.” Nature 404, no. 6775: 293–296. 10749213 10.1038/35005107

[gtc70074-bib-0017] He, K. , X. Zhang , S. Ren , and J. Sun . 2016. “Deep Residual Learning for Image Recognition.” In Proceedings of the IEEE Conference on Computer Vision and Pattern Recognition, 770–778. IEEE.

[gtc70074-bib-0018] He, W. , Y. Wang , L. Cui , R. Su , and L. Wei . 2021. “Learning Embedding Features Based on Multisense‐Scaled Attention Architecture to Improve the Predictive Performance of Anticancer Peptides.” Bioinformatics 37, no. 24: 4684–4693. 34323948 10.1093/bioinformatics/btab560

[gtc70074-bib-0019] Hofacker, I. L. 2003. “Vienna RNA Secondary Structure Server.” Nucleic Acids Research 31, no. 13: 3429–3431. 12824340 10.1093/nar/gkg599 PMC169005

[gtc70074-bib-0020] Hu, B. , L. Zhong , Y. Weng , et al. 2020. “Therapeutic siRNA: State of the Art.” Signal Transduction and Targeted Therapy 5, no. 1: 101. 32561705 10.1038/s41392-020-0207-x PMC7305320

[gtc70074-bib-0021] Huang, J. , F. Wang , E. Argyris , et al. 2007. “Cellular microRNAs Contribute to HIV‐1 Latency in Resting Primary CD4+ T Lymphocytes.” Nature Medicine 13, no. 10: 1241–1247. 10.1038/nm1639 17906637

[gtc70074-bib-0022] Hutvagner, G. , J. McLachlan , A. E. Pasquinelli , É. Bálint , T. Tuschl , and P. D. Zamore . 2001. “A Cellular Function for the RNA‐Interference Enzyme Dicer in the Maturation of the Let‐7 Small Temporal RNA.” Science 293, no. 5531: 834–838. 11452083 10.1126/science.1062961

[gtc70074-bib-0023] Kalayinia, S. , F. Arjmand , M. Maleki , M. Malakootian , and C. P. Singh . 2021. “MicroRNAs: Roles in Cardiovascular Development and Disease.” Cardiovascular Pathology 50: 107296. 33022373 10.1016/j.carpath.2020.107296

[gtc70074-bib-0024] Ketting, R. F. , S. E. Fischer , E. Bernstein , T. Sijen , G. J. Hannon , and R. H. Plasterk . 2001. “Dicer Functions in RNA Interference and in Synthesis of Small RNA Involved in Developmental Timing in *C. elegans* .” Genes & Development 15, no. 20: 2654–2659. 11641272 10.1101/gad.927801 PMC312808

[gtc70074-bib-0025] Kim, V. N. , J. Han , and M. C. Siomi . 2009. “Biogenesis of Small RNAs in Animals.” Nature Reviews Molecular Cell Biology 10, no. 2: 126–139. 19165215 10.1038/nrm2632

[gtc70074-bib-0026] Kimura, M. , S. Kothari , W. Gohir , J. F. Camargo , and S. Husain . 2023. “MicroRNAs in Infectious Diseases: Potential Diagnostic Biomarkers and Therapeutic Targets.” Clinical Microbiology Reviews 36, no. 4: e00015‐23. 37909789 10.1128/cmr.00015-23 PMC10732047

[gtc70074-bib-0027] Knight, S. W. , and B. L. Bass . 2001. “A Role for the RNase III Enzyme DCR‐1 in RNA Interference and Germ Line Development in *Caenorhabditis elegans* .” Science 293, no. 5538: 2269–2271. 11486053 10.1126/science.1062039 PMC1855227

[gtc70074-bib-0028] Lau, P. W. , K. Z. Guiley , N. De , C. S. Potter , B. Carragher , and I. J. MacRae . 2012. “The Molecular Architecture of Human Dicer.” Nature Structural & Molecular Biology 19, no. 4: 436–440. 10.1038/nsmb.2268 PMC3319852 22426548

[gtc70074-bib-0029] Le, T. N. Y. , C. T. Le , and T. A. Nguyen . 2024. “Determinants of Selectivity in the Dicing Mechanism.” Nature Communications 15, no. 1: 8989. 10.1038/s41467-024-53322-1 PMC11487123 39420173

[gtc70074-bib-0030] Lee, R. C. , R. L. Feinbaum , and V. Ambros . 1993. “The *C. elegans* Heterochronic Gene Lin‐4 Encodes Small RNAs With Antisense Complementarity to Lin‐14.” Cell 75, no. 5: 843–854. 8252621 10.1016/0092-8674(93)90529-y

[gtc70074-bib-0031] Lewis, B. P. , I. H. Shih , M. W. Jones‐Rhoades , D. P. Bartel , and C. B. Burge . 2003. “Prediction of Mammalian microRNA Targets.” Cell 115, no. 7: 787–798. 14697198 10.1016/s0092-8674(03)01018-3

[gtc70074-bib-0032] Li, W. , and A. Godzik . 2006. “Cd‐Hit: A Fast Program for Clustering and Comparing Large Sets of Protein or Nucleotide Sequences.” Bioinformatics 22, no. 13: 1658–1659. 16731699 10.1093/bioinformatics/btl158

[gtc70074-bib-0033] Li, Z. , P. Ren , H. Yang , J. Zheng , and F. Bai . 2024. “TEFDTA: A Transformer Encoder and Fingerprint Representation Combined Prediction Method for Bonded and Non‐Bonded Drug–Target Affinities.” Bioinformatics 40, no. 1: btad778. 38141210 10.1093/bioinformatics/btad778 PMC10777355

[gtc70074-bib-0034] Liu, P. , J. Song , C. Y. Lin , and T. Akutsu . 2021. “ReCGBM: A Gradient Boosting‐Based Method for Predicting Human Dicer Cleavage Sites.” BMC Bioinformatics 22, no. 1: 63. 33568063 10.1186/s12859-021-03993-0 PMC7877110

[gtc70074-bib-0066] Loshchilov, I. , and F. Hutter . 2017. “Decoupled Weight Decay Regularization.” arXiv preprint arXiv:1711.05101.

[gtc70074-bib-0035] Lu, J. , G. Getz , E. A. Miska , et al. 2005. “MicroRNA Expression Profiles Classify Human Cancers.” Nature 435, no. 7043: 834–838. 15944708 10.1038/nature03702

[gtc70074-bib-0036] Luo, Q. J. , J. Zhang , P. Li , et al. 2021. “RNA Structure Probing Reveals the Structural Basis of Dicer Binding and Cleavage.” Nature Communications 12, no. 1: 3397. 10.1038/s41467-021-23607-w PMC8184798 34099665

[gtc70074-bib-0037] Maas, A. L. , A. Y. Hannun , and A. Y. Ng . 2013. “Rectifier Nonlinearities Improve Neural Network Acoustic Models.” In Proceedings of the 30th International Conference on Machine Learning, vol. 30, 3.

[gtc70074-bib-0038] MacRae, I. J. , K. Zhou , and J. A. Doudna . 2007. “Structural Determinants of RNA Recognition and Cleavage by Dicer.” Nature Structural & Molecular Biology 14, no. 10: 934–940. 10.1038/nsmb1293 17873886

[gtc70074-bib-0039] MacRae, I. J. , K. Zhou , F. Li , et al. 2006. “Structural Basis for Double‐Stranded RNA Processing by Dicer.” Science 311, no. 5758: 195–198. 16410517 10.1126/science.1121638

[gtc70074-bib-0040] Mu, L. , J. Song , T. Akutsu , and T. Mori . 2024. “DiCleave: A Deep Learning Model for Predicting Human Dicer Cleavage Sites.” BMC Bioinformatics 25, no. 1: 13. 38195423 10.1186/s12859-024-05638-4 PMC10775615

[gtc70074-bib-0041] Nguyen, T. A. , M. H. Jo , Y. G. Choi , et al. 2015. “Functional Anatomy of the Human Microprocessor.” Cell 161, no. 6: 1374–1387. 26027739 10.1016/j.cell.2015.05.010

[gtc70074-bib-0042] Nguyen, T. D. , T. A. Trinh , S. Bao , and T. A. Nguyen . 2022. “Secondary Structure RNA Elements Control the Cleavage Activity of DICER.” Nature Communications 13, no. 1: 2138. 10.1038/s41467-022-29822-3 PMC9018771 35440644

[gtc70074-bib-0043] O'Brien, J. , H. Hayder , Y. Zayed , and C. Peng . 2018. “Overview of microRNA Biogenesis, Mechanisms of Actions, and Circulation.” Frontiers in Endocrinology 9: 402. 30123182 10.3389/fendo.2018.00402 PMC6085463

[gtc70074-bib-0044] Park, J. E. , I. Heo , Y. Tian , et al. 2011. “Dicer Recognizes the 5′ End of RNA for Efficient and Accurate Processing.” Nature 475, no. 7355: 201–205. 21753850 10.1038/nature10198 PMC4693635

[gtc70074-bib-0045] Pauley, K. M. , S. Cha , and E. K. Chan . 2009. “MicroRNA in Autoimmunity and Autoimmune Diseases.” Journal of Autoimmunity 32, no. 3–4: 189–194. 19303254 10.1016/j.jaut.2009.02.012 PMC2717629

[gtc70074-bib-0046] Sardh, E. , P. Harper , M. Balwani , et al. 2019. “Phase 1 Trial of an RNA Interference Therapy for Acute Intermittent Porphyria.” New England Journal of Medicine 380, no. 6: 549–558. 30726693 10.1056/NEJMoa1807838

[gtc70074-bib-0047] Skalsky, R. L. , and B. R. Cullen . 2010. “Viruses, MicroRNAs, and Host Interactions.” Annual Review of Microbiology 64: 123–141. 10.1146/annurev.micro.112408.134243 PMC3621958 20477536

[gtc70074-bib-0048] Small, E. M. , and E. N. Olson . 2011. “Pervasive Roles of microRNAs in Cardiovascular Biology.” Nature 469, no. 7330: 336–342. 21248840 10.1038/nature09783 PMC3073349

[gtc70074-bib-0049] Starega‐Roslan, J. , P. Galka‐Marciniak , and W. J. Krzyzosiak . 2015. “Nucleotide Sequence of miRNA Precursor Contributes to Cleavage Site Selection by Dicer.” Nucleic Acids Research 43, no. 22: 10939–10951. 26424848 10.1093/nar/gkv968 PMC4678860

[gtc70074-bib-0050] Taganov, K. D. , M. P. Boldin , K. J. Chang , and D. Baltimore . 2006. “NF‐κB‐Dependent Induction of MicroRNA miR‐146, an Inhibitor Targeted to Signaling Proteins of Innate Immune Responses.” Proceedings of the National Academy of Sciences of the United States of America 103, no. 33: 12481–12486. 16885212 10.1073/pnas.0605298103 PMC1567904

[gtc70074-bib-0051] Takamizawa, J. , H. Konishi , K. Yanagisawa , et al. 2004. “Reduced Expression of the Let‐7 MicroRNAs in Human Lung Cancers in Association With Shortened Postoperative Survival.” Cancer Research 64, no. 11: 3753–3756. 15172979 10.1158/0008-5472.CAN-04-0637

[gtc70074-bib-0052] Tang, Y. , X. Luo , H. Cui , et al. 2009. “MicroRNA‐146a Contributes to Abnormal Activation of the Type I Interferon Pathway in Human Lupus by Targeting the Key Signaling Proteins.” Arthritis and Rheumatism 60, no. 4: 1065–1075. 19333922 10.1002/art.24436

[gtc70074-bib-0053] Thum, T. , D. Catalucci , and J. Bauersachs . 2008. “MicroRNAs: Novel Regulators in Cardiac Development and Disease.” Cardiovascular Research 79, no. 4: 562–570. 18511432 10.1093/cvr/cvn137

[gtc70074-bib-0054] Vaswani, A. , N. Shazeer , N. Parmar , et al. 2017. “Attention Is All You Need.” In Advances in Neural Information Processing Systems, edited by I. Guyon , U. von Luxburg , S. Bengio , et al., vol. 30. Curran Associates, Inc.

[gtc70074-bib-0055] Vermeulen, A. , L. Behlen , A. Reynolds , et al. 2005. “The Contributions of dsRNA Structure to Dicer Specificity and Efficiency.” RNA 11, no. 5: 674–682. 15811921 10.1261/rna.7272305 PMC1370754

[gtc70074-bib-0056] Weng, Y. , H. Xiao , J. Zhang , X. J. Liang , and Y. Huang . 2019. “RNAi Therapeutic and Its Innovative Biotechnological Evolution.” Biotechnology Advances 37, no. 5: 801–825. 31034960 10.1016/j.biotechadv.2019.04.012

[gtc70074-bib-0057] Wightman, B. , I. Ha , and G. Ruvkun . 1993. “Posttranscriptional Regulation of the Heterochronic Gene Lin‐14 by Lin‐4 Mediates Temporal Pattern Formation in *C. elegans* .” Cell 75, no. 5: 855–862. 8252622 10.1016/0092-8674(93)90530-4

[gtc70074-bib-0058] Yan, L. , M. Liang , X. Hou , et al. 2019. “The Role of MicroRNA‐16 in the Pathogenesis of Autoimmune Diseases: A Comprehensive Review.” Biomedicine & Pharmacotherapy 112: 108583. 30780103 10.1016/j.biopha.2019.01.044

[gtc70074-bib-0059] Yao, D. , B. Li , X. Zhan , X. Zhan , and L. Yu . 2024. “GCNFORMER: Graph Convolutional Network and Transformer for Predicting lncRNA‐Disease Associations.” BMC Bioinformatics 25, no. 1: 5. 38166659 10.1186/s12859-023-05625-1 PMC10763317

[gtc70074-bib-0060] Yoshida, T. , Y. Asano , and K. Ui‐Tei . 2021. “Modulation of MicroRNA Processing by Dicer via Its Associated dsRNA Binding Proteins.” Non‐Coding RNA 7, no. 3: 57. 34564319 10.3390/ncrna7030057 PMC8482068

[gtc70074-bib-0061] Yu, F. , H. Yao , P. Zhu , et al. 2007. “Let‐7 Regulates Self Renewal and Tumorigenicity of Breast Cancer Cells.” Cell 131, no. 6: 1109–1123. 18083101 10.1016/j.cell.2007.10.054

[gtc70074-bib-0062] Zhang, H. , F. A. Kolb , L. Jaskiewicz , E. Westhof , and W. Filipowicz . 2004. “Single Processing Center Models for Human Dicer and Bacterial RNase III.” Cell 118, no. 1: 57–68. 15242644 10.1016/j.cell.2004.06.017

[gtc70074-bib-0063] Zhang, S. , R. Fan , Y. Liu , S. Chen , Q. Liu , and W. Zeng . 2023. “Applications of Transformer‐Based Language Models in Bioinformatics: A Survey.” Bioinformatics Advances 3, no. 1: vbad001. 36845200 10.1093/bioadv/vbad001 PMC9950855

[gtc70074-bib-0064] Zhang, S. , Y. Xu , and Y. Liang . 2024. “TMSC‐m7G: A Transformer Architecture Based on Multi‐Sense‐Scaled Embedding Features and Convolutional Neural Network to Identify RNA N7‐Methylguanosine Sites.” Computational and Structural Biotechnology Journal 23: 129–139. 38089465 10.1016/j.csbj.2023.11.052 PMC10714341

[gtc70074-bib-0065] Zhao, Y. , E. Samal , and D. Srivastava . 2005. “Serum Response Factor Regulates a Muscle‐Specific microRNA That Targets Hand2 During Cardiogenesis.” Nature 436, no. 7048: 214–220. 15951802 10.1038/nature03817

