# Supplementary material for: DiCleavePlus: A Transformer‐Based Model to Detect Human Dicer Cleavage Sites Within Cleavage Patterns
Source: Genes Cells. 2025 Dec 14;31(1):e70074. doi: 10.1111/gtc.70074 (PMC12703071; doi:10.1111/gtc.70074)
Supplement: Supplementary file 1 — Figure S1: Linear representation of human pre‐miRNA hsa‐let‐7a. The blue‐shaded regions correspond to two mature miRNAs, hsa‐let‐7a‐5p, and hsa‐let‐7a‐3p. The position of Dicer cleavage sites (red triangles) and Drosha cleavage sites (blue triangles) is inferred from these mature miRNAs. Red frames highlight 14‐nt Cleavage Patterns in which the Dicer cleavage site is located at the central interval. Figure S2: Workflow for dataset construction. Figure S3: Confusion matrix heatmaps of DiCleavePlus models trained and evaluated on Dataset‐1 with different pattern sizes. (A) DCP‐AFF with 14‐nt patterns; (B) DCP‐Concat with 14‐nt patterns; (C) DCP‐AFF with 18‐nt patterns; and (D) DCP‐Concat with 18‐nt patterns. The x‐axis denotes the predicted class; the y‐axis denotes the true class. Class 0 corresponds to negative patterns (i.e., patterns that do not contain a cleavage site). Figure S4: Data processing workflow of DiCleavePlus. (A) Pre‐miRNA sequences (blue blocks) and their corresponding secondary structures (green blocks) are tokenized into 3‐mers and encoded using 32‐dimensional embeddings. (B) The sequence embeddings are concatenated with the secondary structure embeddings to generate the input features of DiCleavePlus. Figure S5: Architecture of the attentional feature fusion (AFF) block used in this study. The orange module represents the multi‐scale channel attention block. The symbol ⊕ denotes element‐wise addition, and ⊗ indicates element‐wise multiplication. The dashed line indicates the computation of complementary weight (1−w). Figure S6: Heatmap of pairwise sequence similarity matrix among the original 956 pre‐miRNAs. (A) Heatmap of pairwise alignment score matrix computed using the BLASTN scoring scheme. (B) Heatmap of pairwise similarity score matrix. Figure S7: Heatmap of pairwise sequence similarity matrix among pre‐miRNAs after applying the 80% CD‐HIT‐EST threshold. (A) Heatmap of pairwise alignment score matrix computed using the BLASTN scoring schem [file GTC-31-0-s005.pdf]

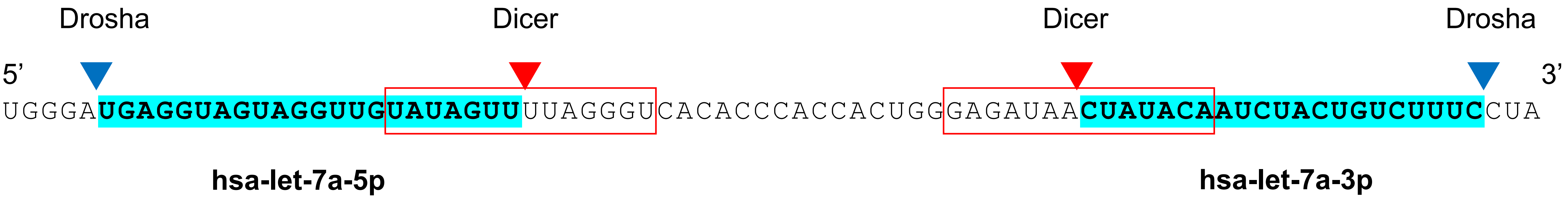


**Sup. Figure S1**

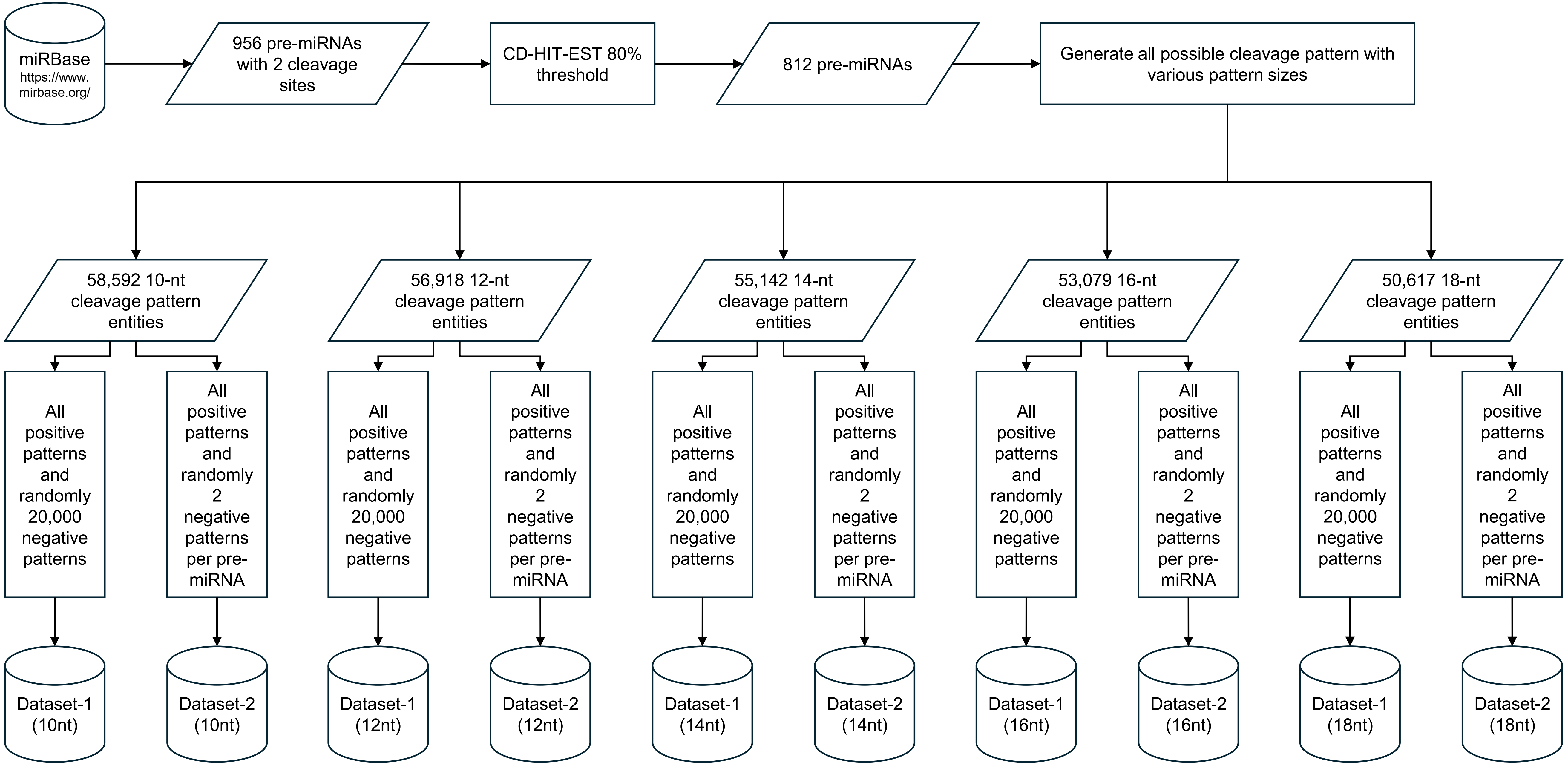


**Sup. Figure S2**

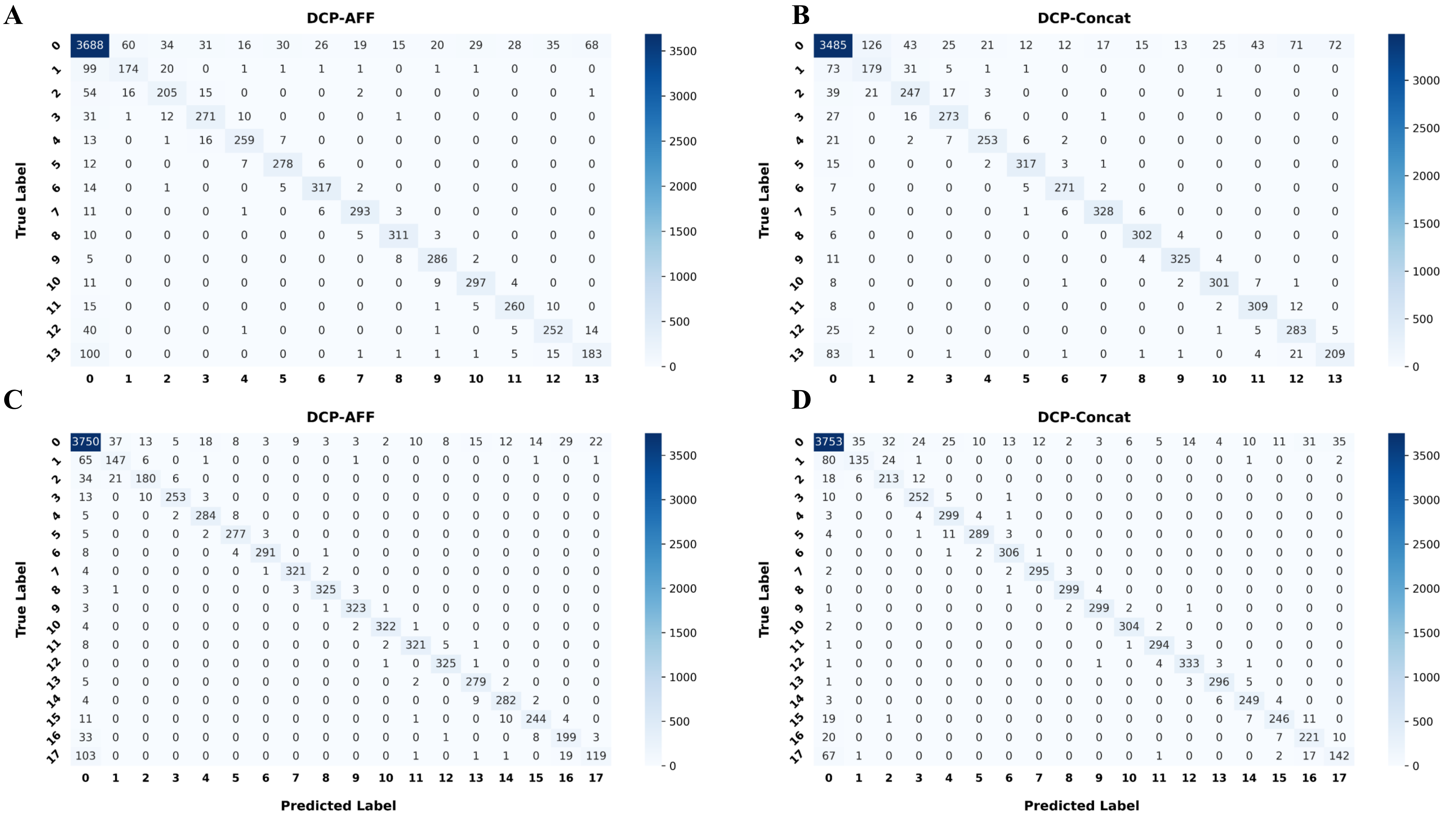


Sup. Figure S3

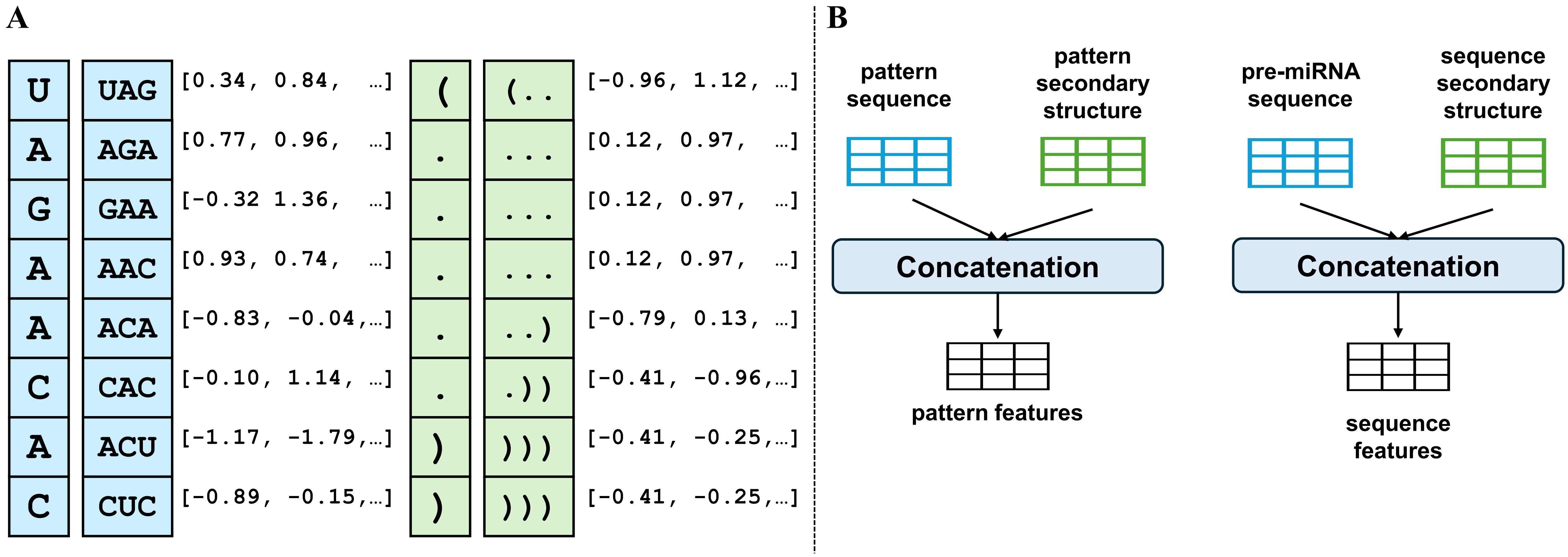


Sup. Figure S4

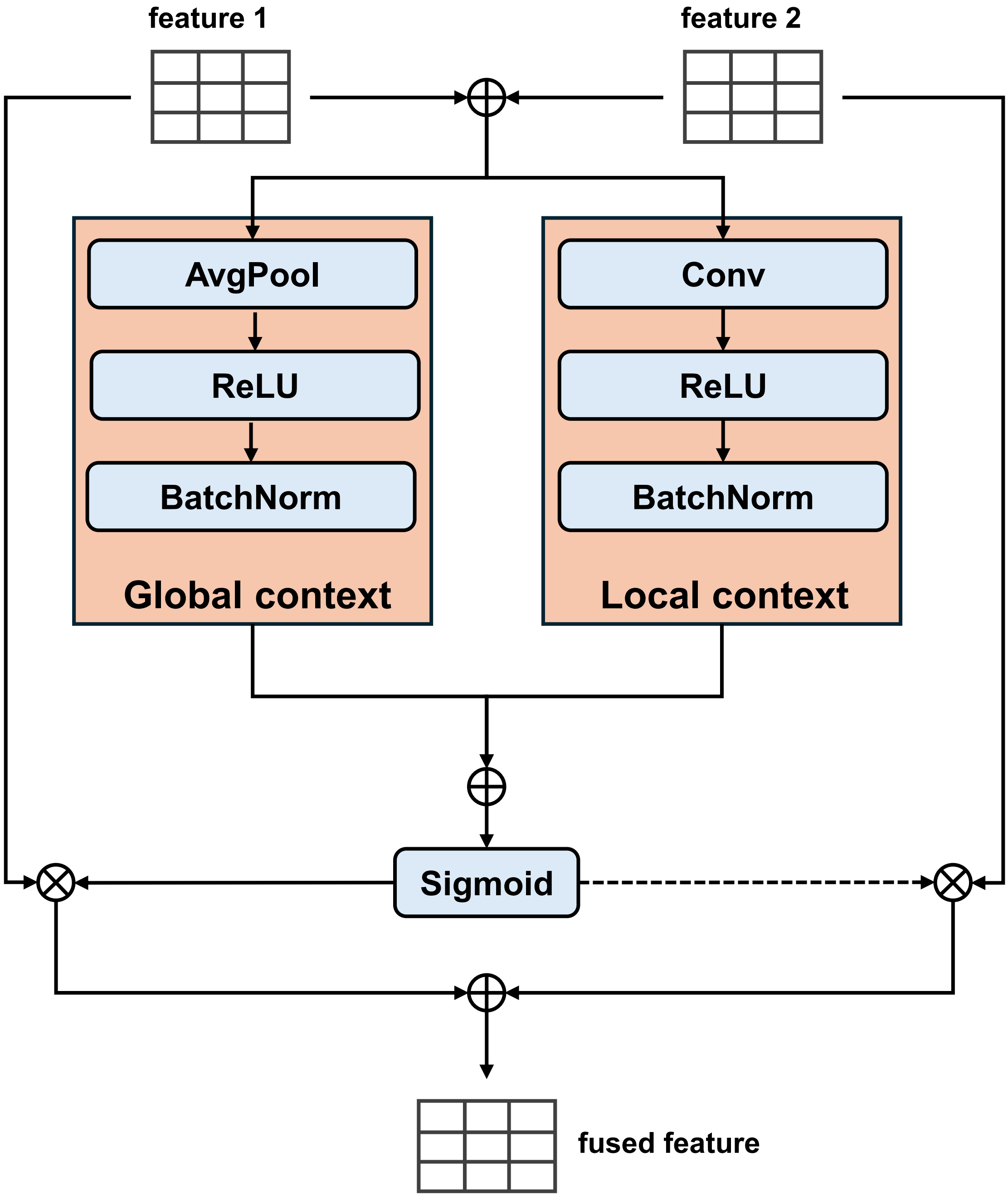


Sup. Figure S5

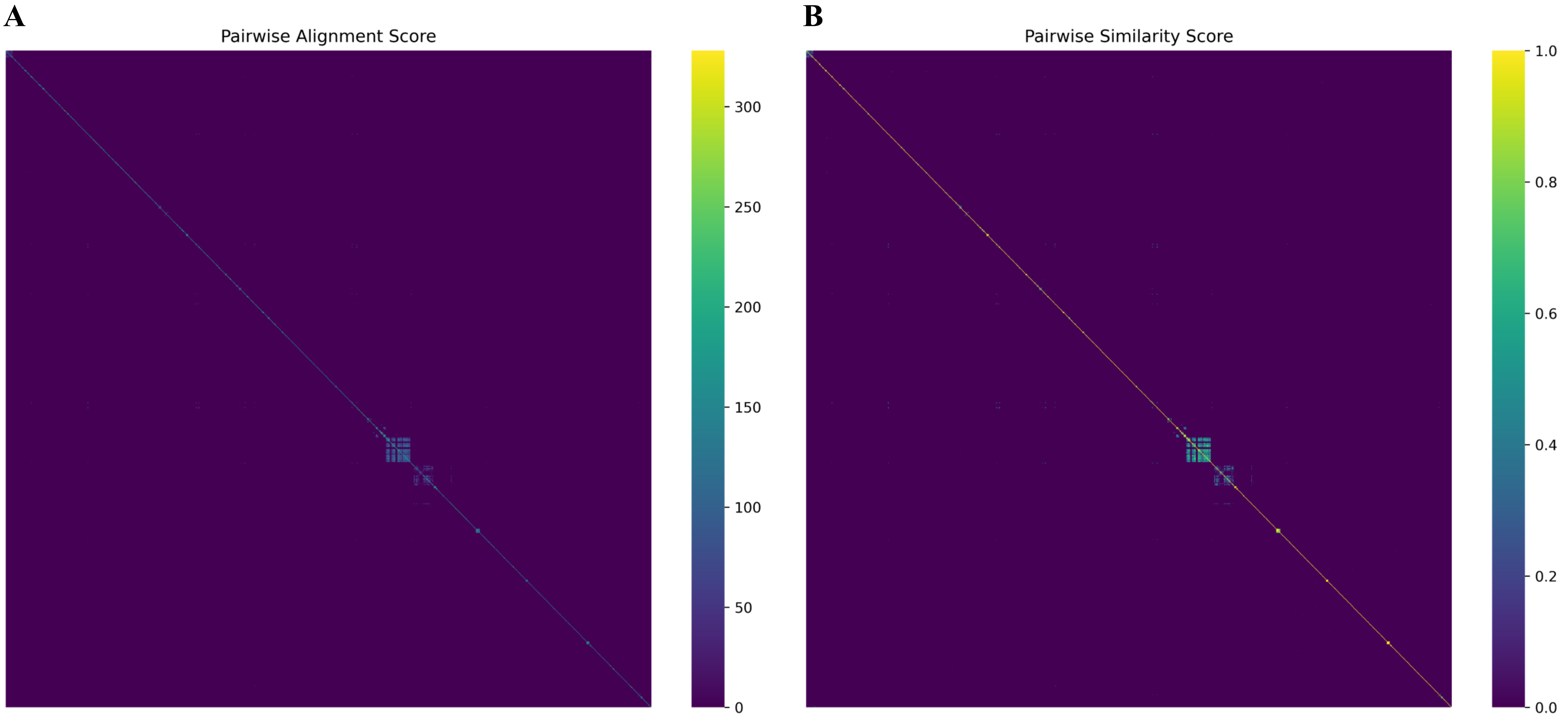


**Sup. Figure S6**

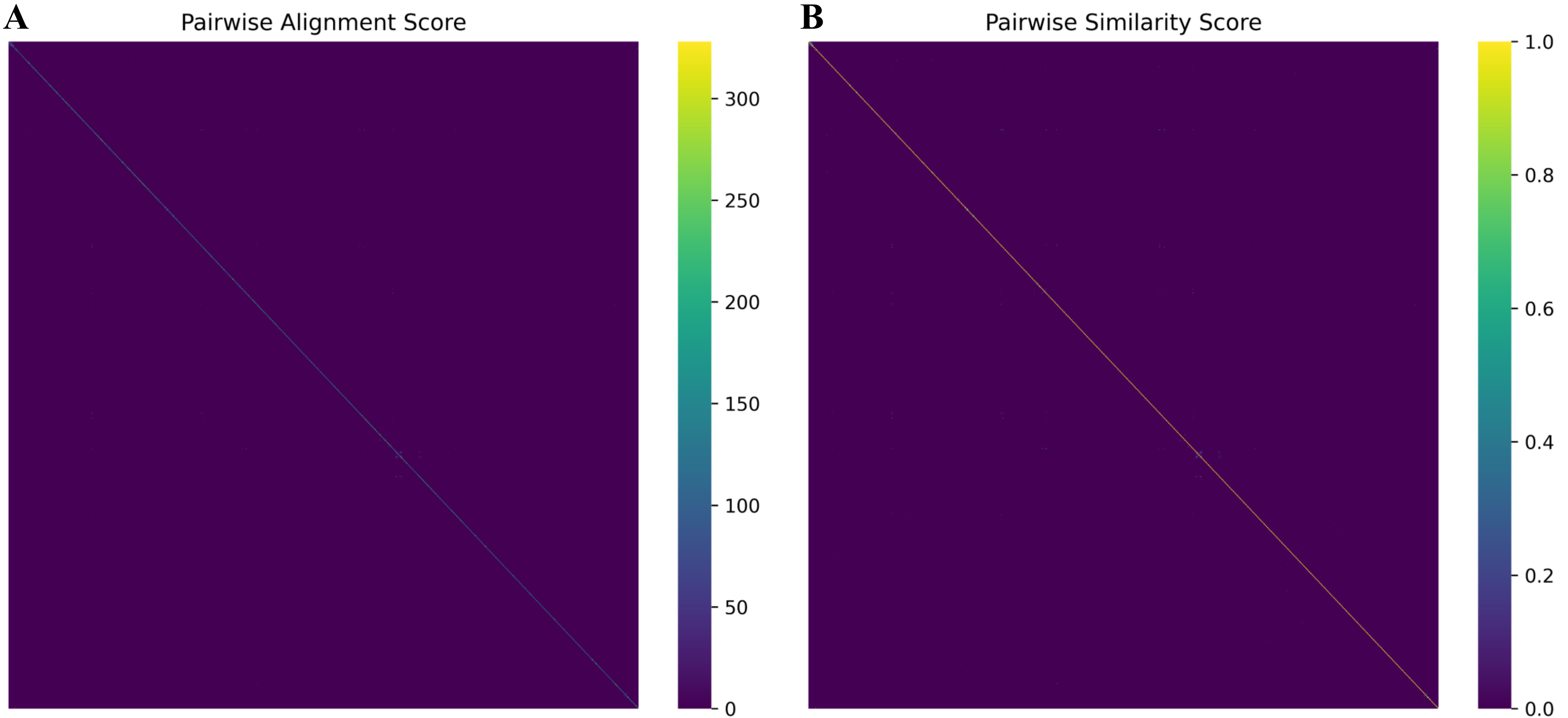


**Sup. Figure S7**

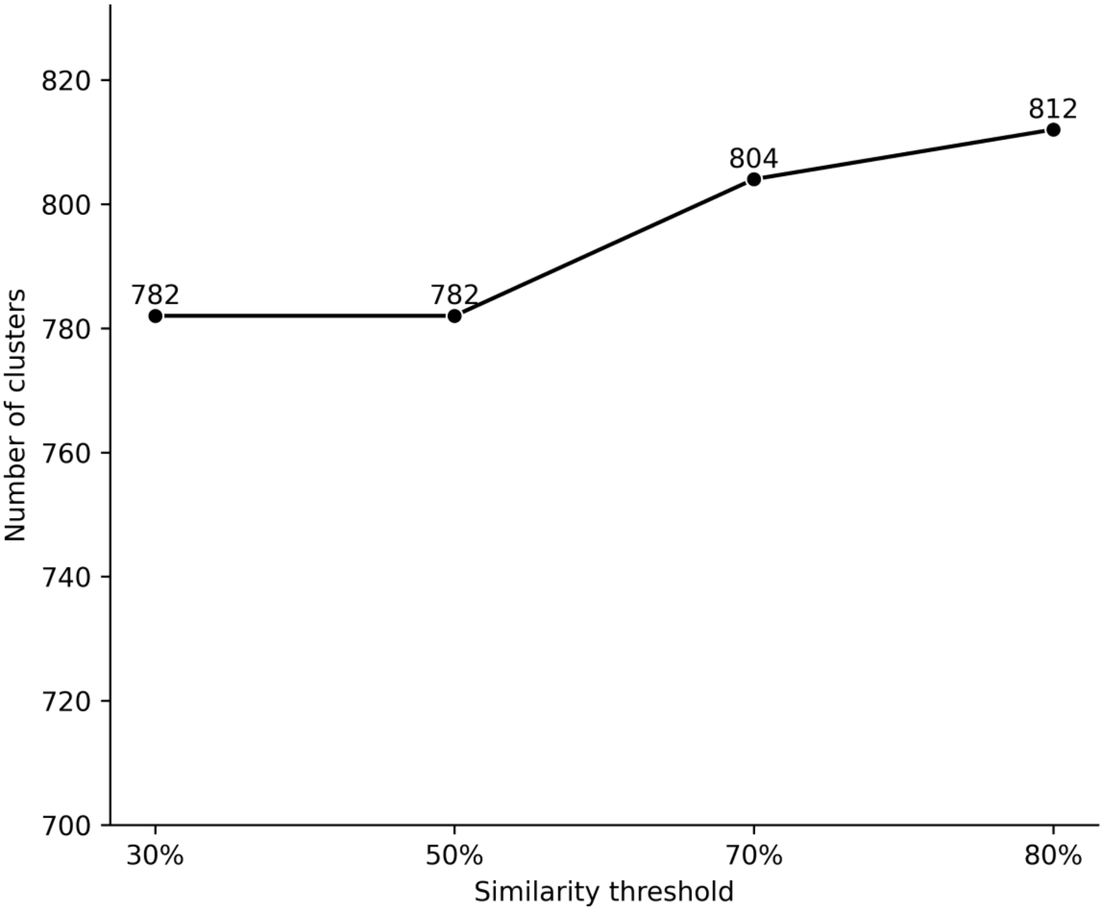


**Sup. Figure S8**
