# Supplementary material for: DiCleavePlus: A Transformer‐Based Model to Detect Human Dicer Cleavage Sites Within Cleavage Patterns
Source: Genes Cells. 2025 Dec 14;31(1):e70074. doi: 10.1111/gtc.70074 (PMC12703071; doi:10.1111/gtc.70074)
Supplement: Supplementary file 2 — Data S1: Figure Legends. [file GTC-31-0-s003.docx]

**Page 1**

**Figure S1: Linear representation of human pre-miRNA hsa-let-7a.** The blue-shaded regions correspond to two mature miRNAs, hsa-let-7a-5p and hsa-let-7a-3p. The position of Dicer cleavage sites (red triangles) and Drosha cleavage sites (blue triangles) are inferred from these mature miRNAs. Red frames highlight 14-nt Cleavage Patterns in which the Dicer cleavage site is located at the central interval.

**Page 2**

**Figure S2: Workflow for dataset construction.**

**Page 3**

**Figure S3: Confusion matrix heatmaps of DiCleavePlus models trained and evaluated on Dataset-1 with different pattern sizes.** A: DCP-AFF with 14-nt patterns; B: DCP-Concat with 14-nt patterns; C: DCP-AFF with 18-nt patterns; D: DCP-Concat with 18 nt patterns. The x-axis denotes the predicted class; the y-axis denotes the true class. Class 0 corresponds to negative patterns (i.e., patterns that do not contain a cleavage site).

**Page 4**

**Figure S4: Data processing workflow of DiCleavePlus.** A: Pre-miRNA sequences (blue blocks) and their corresponding secondary structures (green blocks) are tokenized into 3-mers and encoded using 32-dimensional embeddings. B: The sequence embeddings are concatenated with the secondary structure embeddings to generate the input features of DiCleavePlus.

**Page 5**

**Figure S5: Architecture of attentional feature fusion (AFF) block used in this study.** The orange module represents the multi-scale channel attention block. The symbol ⊕ denotes element-wise addition, and ⊗ indicates element-wise multiplication. The dash line indicates the computation of complementary weight ($1-w$).

**Page 6**

**Figure S6: Heatmap of pairwise sequence similarity matrix among the original 956 pre-miRNAs.** A: Heatmap of pairwise alignment score matrix computed using the BLASTN scoring scheme. B: Heatmap of pairwise similarity score matrix.

**Page 7**

**Figure S7: Heatmap of pairwise sequence similarity matrix among pre-miRNAs after applying the 80% CD-HIT-EST threshold.** A: Heatmap of pairwise alignment score matrix computed using the BLASTN scoring scheme. B: Heatmap of pairwise similarity score matrix.

**Page 8**

**Figure S8: Number of pre-miRNA clusters under different sequence similarity thresholds.** The x-axis represents the similarity threshold used for clustering, and the y-axis represents the corresponding number of pre-miRNA clusters.
