# Supplementary material for: DiCleavePlus: A Transformer‐Based Model to Detect Human Dicer Cleavage Sites Within Cleavage Patterns
Source: Genes Cells. 2025 Dec 14;31(1):e70074. doi: 10.1111/gtc.70074 (PMC12703071; doi:10.1111/gtc.70074)
Supplement: Supplementary file 8 — File S1: Discussion about similarity cutoff threshold. [file GTC-31-0-s007.docx]

**Discussion about similarity cutoff threshold**

In this study, CD-HIT-EST with an 80% sequence similarity threshold was applied to remove redundant pre-miRNA sequences. This threshold has also been empirically adopted in previous studies such as LBSizeCleav (Bao et al., 2016)and DiCleave (Mu et al., 2024). To evaluate whether this cutoff is sufficiently stringent to eliminate highly similar sequences, we conducted a comprehensive analysis of sequence similarity among human pre-miRNAs.

We first investigated the similarity characteristics of the original 956 pre-miRNA sequences. Pairwise alignment scores were computed using the BLASTN scoring scheme (Altschul et al., 1990). Because BLAST alignment scores dependent on both compared sequence length and are not normalized, we converted them into a normalized similarity score ranging from 0 to 1. The pairwise similarity score was defined as:

$${score}_{PS}=\frac{{score}_{PA}}{\min\left( len\left( {seq}_{a} \right),len\left( {seq}_{b} \right) \right)\times S_{match}}$$

where ${score}_{PS}$ denotes pairwise similarity score; ${score}_{PA}$ is the BLAST pairwise alignment score; $len({seq}_{a})$, $len({seq}_{b})$ are the length of two aligned sequences; $S_{match}$ is the score for a nucleotide match under the BLAST scoring scheme, which was set to 2.

As shown in Supplementary Figure S6, most of the 956 pre-miRNA sequences exhibited low pairwise similarity, with only a small fraction displaying similarity scores greater than 0.6. After applying CD-HIT-EST with an 80% similarity threshold, the majority of these highly similar sequences were successfully removed (Supplementary Figure S7), suggesting that this threshold is effective in reducing redundancy.

To further assess the influence of different similarity thresholds, we performed clustering using lower thresholds. Since CD-HIT-EST does not support similarity thresholds below 80%, VSEARCH (Rognes et al., 2016) was employed to cluster sequences at 30%, 50%, and 70% similarity levels. Given the generally low similarity among the original 956 pre-miRNA sequences, clustering results showed minimal differences between the 80% threshold and lower thresholds (Supplementary Figure S8).

Additionally, DiCleavePlus was retrained on datasets constructed using 50% and 70% similarity thresholds. The model achieved comparable performance to that obtained using the 80% threshold (Supplementary Tables S4 and S5), further demonstrating that the 80% cutoff threshold is sufficient for eliminating redundant sequences without sacrificing dataset diversity.

**Supplementary File Reference**

Altschul, S. F., Gish, W., Miller, W., Myers, E. W., & Lipman, D. J. (1990). Basic local alignment search tool. Journal of molecular biology, 215(3), 403-410.

Bao, Y., Hayashida, M., & Akutsu, T. (2016). LBSizeCleav: improved support vector machine (SVM)-based prediction of Dicer cleavage sites using loop/bulge length. *BMC bioinformatics*, *17*(1), 487.

Mu, L., Song, J., Akutsu, T., & Mori, T. (2024). DiCleave: a deep learning model for predicting human Dicer cleavage sites. *BMC bioinformatics*, *25*(1), 13.

Rognes, T., Flouri, T., Nichols, B., Quince, C., & Mahé, F. (2016). VSEARCH: a versatile open source tool for metagenomics. PeerJ, 4, e2584.
